# Coclique level structure for stochastic chemical reaction networks

**DOI:** 10.1007/s00285-025-02261-6

**Published:** 2025-11-10

**Authors:** Simone Bruno, Yi Fu, Felipe A. Campos, Domitilla Del Vecchio, Ruth J. Williams

**Affiliations:** 1 Department of Data Science, Dana-Farber Cancer Institute, 450 Brookline Avenue, Boston, MA 02115 USA; 2 Department of Mechanical Engineering, Massachusetts Institute of Technology, 77 Massachusetts Avenue, Cambridge, MA 02139 USA; 3 Bioinformatics and Systems Biology Program, University of California, San Diego, 9500 Gilman Drive, La Jolla, CA 92093-0112 USA; 4 Department of Mathematics, University of California, San Diego, 9500 Gilman Drive, La Jolla, CA 92093-0112 USA

**Keywords:** Continuous time Markov chain, Model reduction, Coclique level structure, Mean first passage times, 92C40, 92C42, 60J28

## Abstract

Continuous time Markov chains are commonly used as models for the stochastic behavior of chemical reaction networks. More precisely, these Stochastic Chemical Reaction Networks (SCRNs) are frequently used to gain a mechanistic understanding of how chemical reaction rate parameters impact the stochastic behavior of these systems. One property of interest is mean first passage times (MFPTs) between states. However, deriving explicit formulas for MFPTs can be highly complex. In order to address this problem, we first introduce the concept of $coclique\, level\, structure$ and develop theorems to determine whether certain SCRNs have this feature by studying associated graphs. Additionally, we develop an algorithm to identify, under specific assumptions, all possible coclique level structures associated with a given SCRN. Finally, we demonstrate how the presence of such a structure in a SCRN allows us to derive closed form formulas for both upper and lower bounds for the MFPTs. Our methods can be applied to SCRNs taking values in a generic finite state space and can also be applied to models with non-mass-action kinetics. We illustrate our results with examples from the biological areas of epigenetics, neurobiology and ecology.

**Supplementary Information:**

The online version contains supplementary material available at 10.1007/s00285-025-02261-6.

## Introduction

### Overview

Stochastic Chemical Reaction Networks (SCRNs) are a class of continuous time Markov chain models used to describe the stochastic dynamics of a chemical system undergoing a series of reactions that change the numbers of molecules of a finite set of species over time.

These models can be used to conduct theoretical studies in different biological areas such as chromatin regulation (see, for instance, Bruno et al. 2022a), enzymatic kinetics (see, for instance, Kang et al. 2019), and intracellular viral kinetics (see, for instance, Srivastava et al. 2002; Haseltine and Rawlings 2002).

Formulas for mean first passage times (MFPTs) between states of the Markov chains are helpful in studying the stochastic behavior. However, while calculating an explicit formula is relatively straightforward for certain one-dimensional models, such as birth-death processes (see SI—Section S.3), this typically becomes significantly more difficult for higher-dimensional SCRNs. A potential approach to compute MFPTs involves matrix inversion techniques (Bruno et al. 2024). While this method can, in principle, lead to closed-form expressions, it becomes algebraically complex when symbolic parameter dependencies are retained, even for moderate system sizes. As the system size grows, the dimension of the matrix to be inverted increases, leading to higher computational costs and even more complex expressions. In this case, fixing parameter values can help reduce the computational burden, but this also reduces information about how parameters influence the MFPT.

The inability to obtain an explicit analytical expression for the MFPT poses a challenge because having one allows for a clear understanding of how reaction rate parameters affect certain stochastic behavior of the system. One possible approach to overcome and study the effect of parameter variations on system dynamics, without calculating explicit formulae, is to exploit comparison theorems for stochastic processes (see, for instance, Campos et al. 2023). However, this would require applying the theorem, if applicable, to a fixed parameter or combination of parameters of interest, thereby increasing the complexity of the study compared to having an analytical formula that can be directly analyzed.

In this paper, we first introduce the concept of $coclique\, level\, structure$ and develop theorems to determine whether certain SCRNs have this feature by studying associated graphs. We also develop an algorithm to identify, under specific assumptions, all possible coclique level structures associated with such SCRNs. Then, we demonstrate how the presence of such a structure in a SCRN allows us to derive closed form formulas for both upper and lower bounds for MFPTs.

We apply the theoretical tools developed in this paper to multiple examples to illustrate how they can be used to determine coclique level structures, to derive formulas for upper and lower bounds for the MFPTs, and then to understand how key biological parameters affect the stochastic behavior of the system. While our focus is on SCRNs, our definition of coclique level structure is for associated continuous time Markov chains with state spaces that are finite subsets of the non-negative integer orthant and in which the set of all possible transition vectors is finite as well. Consequently, our approach potentially has broader applications to other models that have similar characteristics to SCRNs.

The structure of the paper is the following: we first introduce some background on SCRNs and mean first passage times (Sect. 2). We then introduce the notion of coclique level structure (Sect. 3) and describe the main theoretical tools developed in this paper (Sect. 4). Finally, we apply our results to multiple examples (Sect. 5) and present some concluding remarks (Sect. 6).

### Related work

In this paper, we introduce the concept of coclique level structure for suitable SCRNs, which will be a partition of the finite state space of an associated continuous time Markov chain, in which the sets in the partition are level sets of a linear function *L* on the state space and they are cocliques in the sense of graph theory (van Mieghem 2010; Kelsey and Roney-Dougal 2022), i.e., there are no direct transitions between states within a set in the partition. To the best of our knowledge, this concept of coclique level structure has not been previously used to derive a reduced stochastic process. Here, we apply the function *L* to a continuous time Markov chain, and analyze the result to estimate MFPTs for SCRNs. Previous theoretical tools developed to evaluate upper and lower bounds for MFPTs are mostly suitable for computational studies (Gaver et al. 1984) and often focus on specific models, such as imprecise birth-death chains (Lopatatzidis et al. 2017) or population continuous time Markov chains (Backenköhler et al. 2020). In contrast to these existing works, the theoretical tools that we develop enable the derivation of closed form formulas for MFPT bounds, making them suitable for analytical analysis.

### Terminology and notation

Denote the set of integers by $\mathbb {Z}$. For an integer $d \ge 2$, we denote by $\mathbb {Z}^d$ the set of *d*-dimensional vectors with entries in $\mathbb {Z}$. Denote by $\mathbb {Z}_+ = \{0,1,2, \ldots \}$, the set of non-negative integers. For an integer $d \ge 2$, we denote by $\mathbb {Z}_+^d$ the set of *d*-dimensional vectors with entries in $\mathbb {Z}_+$. We denote by $0\!\!0$, respectively, $1\!\!1$ a vector of any dimension where all entries are 0’s, respectively, 1’s. The size of $0\!\!0$ or $1\!\!1$ will be understood from the context. The set of real numbers will be denoted by $\mathbb {R}$, $\mathbb {R}_+=[0,\infty )$, and *d*-dimensional Euclidean space will be denoted by $\mathbb {R}^d$ for $d \ge 2$. For integers $m,n \ge 1$, the set of $m \times n$ matrices with real-valued entries will be denoted by $\mathbb {R}^{m \times n}$. For a matrix $A \in \mathbb {R}^{m \times n}$, we denote the kernel of *A* by . Vectors are column vectors unless indicated otherwise and a superscript of *T* will denote the transpose of a vector or matrix.

## Stochastic chemical reaction networks (SCRNs)

In this section, we provide basic definitions for a class of continuous time Markov chains called Stochastic Chemical Reaction Networks (SCRNs). The reader is referred to Anderson and Kurtz (2015) for a more in depth introduction to this subject. The models considered in our examples will be SCRNs and the state space of all of our models will be finite.

We assume there is a finite non-empty set $\mathpzc{\scriptstyle S}\:= \{\textrm{S}_1,\ldots ,\textrm{S}_d\}$ of *d* **species**, and a finite non-empty set $\mathpzc{\scriptstyle R}\:\subseteq \mathbb {Z}_+^d \times \mathbb {Z}_+^d$ that represents chemical **reactions**. We assume that $(w,w) \notin\mathpzc{\scriptstyle R}\:$ for every $w \in \mathbb {Z}^d_+$. The set $\mathpzc{\scriptstyle S}\:\:$ represents *d* different molecular species in a system subject to reactions $\mathpzc{\scriptstyle R}\:$ which change the number of molecules of some species. For each $(v^{-},v^+) \in \mathpzc{\scriptstyle R}\:$, the *d*-dimensional vector $v^{-}$ (the **reactant vector**) counts how many molecules of each species are consumed in the reaction, while $v^{+}$ (the **product vector**) counts how many molecules of each species are produced. The associated reaction is usually written as

2.1 $$\begin{aligned} \sum _{i=1}^d (v^{-})_{i}\textrm{S}_i \longrightarrow \sum _{i=1}^d (v^{+})_{i}\textrm{S}_i. \end{aligned}$$

To avoid the use of unnecessary species, we will assume that for each $1 \le i \le d$, there exists a vector $w=(w_1, \ldots ,w_d)^T \in \mathbb {Z}_+^d$ with $w_i >0$ such that ($w, v$) or ($v, w$) is in$\:\mathpzc{\scriptstyle R}\:$ for some $v \in \mathbb {Z}^d_+$, i.e., each species is either a reactant or a product in some reaction. The net change in the quantity of molecules of each species due to a reaction $(v^{-},v^{+}) \in \mathpzc{\scriptstyle R}\:$ is described by $v^{+}-v^{-}$ and it is called the associated **reaction vector**. We denote the set of reaction vectors by $\mathcal {V}:= \{ v \in \mathbb {Z}^d : v = v^{+}- v^{-} \text { for some } (v^{-},v^{+}) \in\mathpzc{\scriptstyle R}\:\}$; we let $n := |\mathcal {V}|$, the size of $\mathcal {V}$; and we enumerate the members of $\mathcal {V}$ as $\{v_1,\ldots ,v_n\}$. Note that $\mathcal {V}$ does not contain the zero vector because $\mathpzc{\scriptstyle R}\:$ has no elements of the form ($w, w$). Different reactions might have the same reaction vector. For each $v_k \in \mathcal {V}$ we consider the set$\mathpzc{\scriptstyle R}\:_{v_k}:= \{(v^{-},v^{+}) \in \mathpzc{\scriptstyle R}\:: v_k =v^{+}-v^{-} \}$. The matrix $S \in \mathbb {R}^{d \times n}$ whose columns are the elements in $\mathcal {V}$ will be called the **stoichiometric matrix**.^1^ In addition, we define a **conservation vector** $m$ (if there is one) as a *d*-dimensional non-zero vector such that $m^TS=0$ and we say that the conservation vector is *unique* if *m* is unique, up to multiplication by a scalar.

Consider sets of species $\mathpzc{\scriptstyle S}\:\:$ and reactions $\mathpzc{\scriptstyle R}\:$, a non-empty set $\mathcal {X}\subseteq \mathbb {Z}^d_+$, and a collection of functions $\Lambda = \{\Lambda _{(v^{-},v^{+})}:\mathcal {X}\longrightarrow \mathbb {R}_+\}_{(v^{-},v^{+}) \in \mathpzc{\scriptstyle R}}\:$ such that for each $x \in \mathcal {X}$ and $(v^{-},v^{+}) \in\mathpzc{\scriptstyle R}\:$, if $x+v^{+}-v^{-} \notin \mathcal {X}$, then $\Lambda _{(v^{-},v^{+})}(x)=0$. Now, for $1 \le k \le n$, define

2.2 $$\begin{aligned} \Upsilon _k(x):= \sum _{(v^{-},v^{+}) \in\mathpzc{\scriptstyle R}\:_{v_k}} \Lambda _{(v^{-},v^{+})}(x). \end{aligned}$$

Note that for each $x \in \mathcal {X}$ and $1 \le k \le n$, if $x +v_k \notin \mathcal {X}$, then $\Upsilon _k(x) = 0$. The functions $\{\Lambda _{(v^{-},v^{+})}:\mathcal {X}\longrightarrow \mathbb {R}_+\}_{(v^{-},v^{+}) \in \mathpzc{\scriptstyle R}}\:$ are called **propensity** or **intensity** functions. A common form for the propensity functions is the following, which is associated with **mass action kinetics**:

2.3 $$\begin{aligned} \Lambda _{(v^{-},v^{+})}(x) = \kappa _{(v^{-},v^{+})}\prod _{i=1}^{d}(x_i)_{(v^{-})_i}, \end{aligned}$$

where $$\{\kappa _{(v^{-},v^{+})}\}_{(v^{-},v^{+}) \in \mathpzc{\scriptstyle R}}\:$$ are non-negative constants and for $$m,\ell \in \mathbb {Z}_+$$, the quantity $$(m)_\ell$$ is the falling factorial, i.e., $$(m)_0 := 1$$ and $$(m)_\ell := m(m-1)\ldots$$$$ (m-\ell +1)$$.

A **stochastic chemical reaction network (SCRN)** (associated with $(\mathpzc{\scriptstyle S}\:,\mathpzc{\scriptstyle R}\:,\mathcal {X},\Lambda )$) is a continuous time Markov chain *X* with state space $\mathcal {X}$ and infinitesimal generator $Q$ given for $x,y \in \mathcal {X}$ by

2.4 $$\begin{aligned} Q_{x,y} = {\left\{ \begin{array}{ll} \Upsilon _k(x) & \text { if } y-x = v_k \text { for some } 1 \le k \le n, \\ - \sum _{k=1}^n\Upsilon _k(x) & \text { if } y = x, \\ 0 & \text { otherwise.} \end{array}\right. } \end{aligned}$$

If a SCRN associated with $(\mathpzc{\scriptstyle S}\:,\mathpzc{\scriptstyle R}\:,\mathcal {X},\Lambda )$ has a conservation vector $m \ne 0$ and $m^TX(0)=x_{\textrm{tot}}$ for some integer $x_{\textrm{tot}}\ge 0$, then $m^TX(t)=x_{\textrm{tot}}$ for every $t \ge 0$. Consequently, we can reduce the dimension of the continuous time Markov chain describing the system by one. In this paper, we will initially be considering SCRNs for which $m=(1,\ldots ,1)^T$ is a conservation vector. Then, the projected process ${\check{X}}=(X_1,\ldots ,X_{d-1})^T$ is again a continuous time Markov chain with finite state space ${\check{\mathcal {X}}}=\{(x_1,\ldots ,x_{d-1})^T \in \mathbb {Z}^{d-1}_+ : (x_1,\ldots ,x_{d-1},x_{\textrm{tot}} - \sum _{i=1}^{d-1} x_i)^T \in \mathcal {X}\}$. We denote its infinitesimal generator by ${\check{Q}}$. We will assume that $|{\check{\mathcal {X}}}| > 1$. Let $\mathcal {B}$ be a nonempty subset of ${\check{\mathcal {X}}}$ such that $\mathcal {B}\ne {\check{\mathcal {X}}}$, and let

2.5 $$\begin{aligned} \tau _\mathcal {B}:= \inf \{t \ge 0: {\check{X}}(t) \in \mathcal {B}\}. \end{aligned}$$

Then, the **mean first passage time (MFPT)** (for ${\check{X}}$) from $x \in {\check{\mathcal {X}}}$ to $\mathcal {B}$ can be defined as

2.6 $$\begin{aligned} h_{x,\mathcal {B}}=\mathbb {E}[\tau _\mathcal {B}\,|\, {\check{X}}(0) = x]. \end{aligned}$$

If $\mathcal {B}= \{y\}$ for some $y \in {\check{\mathcal {X}}}$, we use the notation $h_{x,y}:= h_{x,\{y\}}$. After considering this simple projection first, later on in Sect. 4.4, we will consider the situation where there are $p>1$ linearly independent conservation vectors for a SCRN. Then, we can reduce the dimension of the Markov chain *X* by $p$, in which case we also denote the projected process by ${\check{X}}$, its state space by ${\check{\mathcal {X}}} \subset \mathbb {Z}^{d-p}_+$ and its infinitesimal generator by ${\check{Q}}$. The mean first passage time for ${\check{X}}$ is then defined by (2.5)–(2.6).

## Coclique level structure for SCRNs

In this section, we define the notion of $coclique\, level\, structure$ for SCRNs and introduce some useful assumptions, definitions, and lemmas that we use in this paper.

First, consider a SCRN, as defined in Sect. 2, and assume the following:

### Assumption 3.1

Assume that $d\ge 2$. For each $v \in \mathcal {V}= \{v_1,\dots ,v_n\}$, the entries of the reaction vector $v$ consist of $d-2$ zeros, a single one and a single minus one, in any order.

When satisfied, this assumption implies that the net change in the number of species stemming from a reaction consists of consuming one molecule of a given species to produce a molecule of a different species. For example, when $d=3$, reactions satisfying this assumption include $\textrm{S}_1 \longrightarrow \textrm{S}_2$ and $\textrm{S}_1 + \textrm{S}_3 \longrightarrow \textrm{S}_2 + \textrm{S}_3$, both of which have associated reaction vector $(-1,1,0)^T$.

We consider a directed graph $\mathcal {G}$ associated with a SCRN satisfying Assumption 3.1, in which the vertices represent the species of the system (we will label these vertices $1,\dots ,d$ or $\textrm{S}_1,\dots ,\textrm{S}_d$) and the directed edges between vertices represent the reaction vectors $\{v_1,\dots ,v_n\}$. In this graph, an edge $e_k=(i,j)$, with $k\in \{1,2,\dots ,n\}$ and $i \ne j$, exists if and only if there is a reaction vector $v_k\in \mathcal {V}$ where $v_k(i) =-1$, $v_k(j)=1$, $v_k(\ell ) =0$ for $\ell \notin \{i,j\}$. Note that, if we have two edges $e_{k}=(i,j)$ and $e_{k'}=(j,i)$, with $i \ne j$, then $v_{k}=-v_{k'}$.

Before introducing the definition of coclique level structure, we provide some useful definitions and lemmas that we will use in this section. Given the directed graph $\mathcal {G}$, a **coclique** is a non-empty set of vertices such that any two vertices in the set do not have a direct edge between them. The graph $\mathcal {G}$ is **bipartite** if the vertex set can be partitioned into two non-empty cocliques. The directed graph $\mathcal {G}$ is **weakly connected** if the underlying undirected graph is connected. Note that this implies $n\ge d-1$. In this section, we will focus on SCRNs whose associated graphs $\mathcal {G}$ are weakly connected, and we will prove results for such SCRNs in Sects. 4.1–4.3.

For a SCRN whose associated graph $\mathcal {G}$ is not weakly connected, we can decompose $\mathcal {G}$ into finitely many disjoint **weakly connected components**. Each of the weakly connected components of $\mathcal {G}$ is a weakly connected graph and there are no edges between different weakly connected components. In Sect. 4.4, we will show how to leverage the results in Sects. 4.1–4.3 for the single weakly connected component case to treat the general case where $\mathcal {G}$ is a finite disjoint union of weakly connected components.

### Lemma 3.1

*Consider a SCRN satisfying Assumption* 3.1*with* $d \ge 2$ *species*, $\mathpzc{\scriptstyle S}\:= \{\textrm{S}_1$, $\ldots ,\textrm{S}_d\}$, *and* *n* *reaction vectors*, $\mathcal {V}=\{v_1,\ldots ,v_n\}$. *Assume that the associated graph* $\mathcal {G}$ *is weakly connected. Then, the rank of the associated stoichiometric matrix* *S*, *whose columns are given by the elements* *of* $\mathcal {V}$, *is equal to* $d-1$. *Furthermore,* $(1,\ldots ,1)^T$ *is the unique (up to scalar multiplication) conservation vector associated with the stoichiometric matrix* *S*.

The proof of Lemma 3.1 is given in SI—Section S.1.

### Remark 3.1

If each vertex in $\mathcal {G}$ is regarded as a species and each edge in $\mathcal {G}$ is regarded as a reaction, then $\mathcal {G}$ can be interpreted as a chemical reaction network in the sense of Feinberg (1986) (where the complexes in this network are simply the species). For this, one can determine its deficiency $\delta =d-\ell -s$, where *d* is the number of complexes (which is the number of species in this case), $\ell$ is the number of weakly connected components in $\mathcal {G}$ and *s* is the rank of the stoichiometric matrix *S* associated with $\mathcal {G}$. One can see by Lemma 3.1 that, when $\mathcal {G}$ is weakly connected, the chemical reaction network associated with $\mathcal {G}$ has deficiency zero.^2^

Under the assumptions of Lemma 3.1, we can introduce a **projected continuous time Markov chain** ${\check{X}}=\{{\check{X}}(t):\, t \ge 0\}$ in which the state $\check{x}$ represents the number of molecules of the first $d-1$ species, that is, $\check{x}=(x_1,\dots ,x_{d-1})^T$. Note that the choice to express $x_d$ as a function of the other $x_i$ with $i\in \{1,\dots ,d-1\}$ is without loss of generality, since the labeling of the species can always be reordered so that the species chosen to be expressed as a function of the others is the last one. The process ${\check{X}}$ is a continuous time Markov chain defined on the finite state space

3.1 $$ \begin{aligned} {\check{\mathcal {X}}}&:=\left\{\vphantom{\left( x_1,\dots ,x{d-1},x_{\textrm{tot}}-\sum_{i=1}^{d-1}x_i\right) ^T} {\check{x}}=(x_1,\dots ,x_{d-1})^T\in \mathbb {Z}_{+}^{d-1}:\right.\\ &\quad\quad\quad\quad\quad\quad\quad \left. \left( x_1,\dots ,x_{d-1},x_{\textrm{tot}}-\sum_{i=1}^{d-1}x_i\right) ^T \in \mathcal {X}\right\} \\&\,\subset \left\{ {\check{x}}=(x_1,\dots ,x_{d-1})^T\in \mathbb {Z}_{+}^{d-1}:x_1+\dots +x_{d-1}\le x_{\textrm{tot}}\right\} ,  \end{aligned}$$

where $x_{\textrm{tot}}=\sum _{i=1}^{d}X_i(0)$. We will assume that $|{\check{\mathcal {X}}}| > 1$, and the infinitesimal generator of ${\check{X}}$ will be denoted by $\check{Q}$.

We now introduce the definitions of coclique level function and coclique level structure. A **coclique level function** for ${\check{X}}$ is a linear function $L: \mathbb {Z}^{d-1} \rightarrow \mathbb {Z}$ such that for each $k= 1,\dots ,n$,

3.2 $$\begin{aligned} L(\check{v}_k) \in \{-1,+1\}, \end{aligned}$$

where $\check{v}_k\in \mathbb {Z}^{d-1}$ is the vector obtained from $v_k$ by removing the last element. If such an *L* exists, it can be written as

3.3 $$\begin{aligned} L(x) = b^T x\,\, \textrm{for}\, x \in \mathbb {Z}^{d-1}\, \mathrm {and\, some}\, b \in \mathbb {Z}^{d-1}, \end{aligned}$$

where, upon partitioning the set of edges of the associated graph $\mathcal {G}$ into two disjoint subsets $\mathcal {E}_+=\{e_k:L(\check{v}_k)=1\}$ and $\mathcal {E}_-=\{e_k:L(\check{v}_k)=-1\}$ (where one of these may be empty), the vector $b=(b_1,\dots ,b_{d-1})^T$ solves the system of equations

3.4 $$\begin{aligned} \sum _{i=1}^{d-1}b_{i} \check{v}_k(i)={\left\{ \begin{array}{ll} +1\,\,\,\text {if }\, e_k \in \mathcal {E}_+\\ -1\,\,\,\text {if }\, e_k \in \mathcal {E}_-\\ \end{array}\right. }\,\,\,\,\, \text {for } k=1,\dots ,n. \end{aligned}$$

Finally, for a coclique level function *L*, the (ordered) partition $\{\mathcal {L}_{\ell }, \dots , \mathcal {L}_u \}$, with

3.5 $$\begin{aligned} \mathcal {L}_z:=\{x \in \check{\mathcal {X}}:\, L(x)=z\}\, \textrm{for}\, z=\ell ,\ell +1,\dots ,u-1,u, \end{aligned}$$

is called a **coclique level structure** for ${\check{X}}$, with

3.6 $$\begin{aligned} \ell =\min \{L(x):x \in {\check{\mathcal {X}}}\},\quad \quad u=\max \{L(x):x \in {\check{\mathcal {X}}}\}. \end{aligned}$$

The sets $\mathcal {L}_{\ell }, \dots , \mathcal {L}_u$ are cocliques in the Markov chain graph for ${\check{X}}$ which consists of states of ${\check{\mathcal {X}}}$ with edges given by $\{({\check{x}}, {\check{x}}+\check{v}_k):k=1,\dots ,n,\,\, {\check{x}}\in {\check{\mathcal {X}}},\,\, {\check{x}}+\check{v}_k\in {\check{\mathcal {X}}}\}$.

### Lemma 3.2

*Consider a SCRN satisfying the assumptions in Lemma* 3.1. *If we partition the set of edges of the associated graph* $\mathcal {G}$ *into two disjoint subsets* $\mathcal {E}_+$ *and* $\mathcal {E}_-$, *where one of these may be empty, then, for this partition* $\{\mathcal {E}_+,\mathcal {E}_-\}$, *the system* (3.4) *admits either zero or one solution, and the solution, if one exists, has integer entries.*

### Proof

Let $\check{S}\in \mathbb {Z}^{(d-1) \times n}$ be the first $(d-1)$ rows of the stoichiometric matrix *S* associated with the SCRN. Then, the system (3.4) can be re-written in matrix–vector form as

3.7 $$\begin{aligned} \check{S}^T b = w, \end{aligned}$$

where $w_k$ is $+1$ or $-1$ depending on whether $e_k \in \mathcal {E}_+$ or $e_k \in \mathcal {E}_-$, respectively, for $k=1,\dots ,n$. By Lemma 3.1, we know that , and thus the last row of *S* is a linear combination of the first $(d-1)$ rows of *S*. This means that removing the last row of *S* does not affect its rank, and thus ${{\,\textrm{rank}\,}}\left( \check{S}^T\right) = d-1$. Hence, if $w$ is in the range of $\check{S}^T$ the system (3.4) admits a unique solution, while if it is not in the range, the system does not admit a solution.

Suppose that (3.4) admits a solution $b \in \mathbb {R}^{d-1}$. Since $\mathcal {G}$ is weakly connected, there is an edge connecting the vertex *d* with another vertex in $\mathcal {G}$, say vertex *i*. This means that there exists $v_k \in \mathcal {V}$ such that $v_k(i)=-v_k(d) \in \{-1,1\}$ and $v_k(\ell )=0$ for $\ell \in \{1,2,\dots ,d-1\} \setminus \{i\}$. Then, since $b \in \mathbb {R}^{d-1}$ solves the $k^{th}$ equation of (3.4), we have that $|b_i|=1$, which means $b_i \in \mathbb {Z}$. Using similar logic, for a vertex $j \notin \{i,d\}$ in $\mathcal {G}$ such that there is an edge between *j* and $\{i,d\}$, we have that either $|b_i-b_j|=1$ or $|b_j|=1$, and thus $b_j \in \mathbb {Z}$. Since $\mathcal {G}$ is weakly-connected and has finitely many vertices, we can iteratively show that all of the entries of *b* have integer values.$\square$

We use the following example to illustrate our theory in a simple context before giving more complex examples in Sect. 5.

**Fig. 1:**
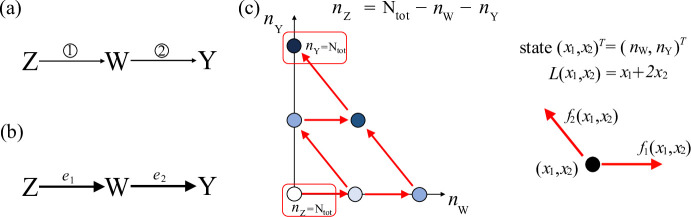
Three-species cascade motif: reaction diagram, graph $\mathcal {G}$ and associated Markov chain (Example 3.1). **a** Chemical reaction system diagram. The numbers on the arrows correspond to the reactions associated with the arrows as described in (3.8) in the main text. **b** Graph $\mathcal {G}$ associated with the chemical reaction system in panel (a). **c** State space and transitions for the projected continuous time Markov chain ${\check{X}}=\{(X_1(t),X_2(t))^T:\, t \ge 0\}$, which keeps track of $(n_{\textrm{W}},n_{\textrm{Y}})$ through time. Here, we consider $\mathrm {N_{tot}}=2$ and we use dots to represent the states, and red arrows to represent transitions. Additionally, we use shades of blue to distinguish the level to which each state belongs. The function $L(x_1,x_2)$ associated with the coclique level structure is $L(x_1,x_2)=x_1+2x_2$. The rates associated with the one-step transitions for the projected Markov chain ${\check{X}}$ are given in (3.10)

### Example 3.1

Consider the following SCRN with mass action kinetics, involving three species and two irreversible reactions:

3.8 $$\begin{aligned} \begin{aligned}&{\textcircled {\small 1}}\,\textrm{Z}{\mathop {\longrightarrow }\limits ^{\alpha }} \textrm{W}, \quad {\textcircled {\small 2}}\,\textrm{W}{\mathop {\longrightarrow }\limits ^{\beta }} \textrm{Y}, \end{aligned} \end{aligned}$$

in which $\alpha , \beta > 0$. The diagram of this SCRN is shown in Fig. 1a. Let the species vector be $x = (n_{\textrm{W}}, n_{\textrm{Y}}, n_{\textrm{Z}})$, where $n_{\textrm{W}}$, $n_{\textrm{Y}}$, $n_{\textrm{Z}}$ denote the number of molecules of species W, Y, Z, respectively. The total number of molecules in this system is conserved, that is, $n_{\textrm{Z}} + n_{\textrm{W}} + n_{\textrm{Y}} = \text {N}_{\text {tot}}$. The reaction vectors associated with this SCRN are $v_1 = (1, 0, -1)^T$ and $v_2 = (-1, 1, 0)^T$. Then, Assumption 3.1 is satisfied, and the associated graph $\mathcal {G}$ is shown in Fig. 1b, which is weakly connected. By Lemma 3.1, our SCRN has a unique conservation vector $m = (1,1,1)^T$, and then we can introduce a projected continuous time Markov chain ${\check{X}}=\{ (X_1(t),X_2(t))^T:\, t \ge 0\}$, which keeps track of $(n_{\textrm{W}},n_{\textrm{Y}})$ through time. Since the total number of molecules $\text {N}_{\text {tot}}$ is conserved, the state space is ${\check{\mathcal {X}}}= \{{\check{x}}=(x_1,x_2)^T \in \mathbb {Z}_+^2 :\, x_1 + x_2 \le \text {N}_{\text {tot}}\}$. The potential one-step transitions for $\check{X}$ from $x \in {\check{\mathcal {X}}}$ are illustrated in Fig. 1c (for $\text {N}_{\text {tot}}=2$), where the associated transition vectors are

3.9 $$\begin{aligned} \check{v}_1=(1,0)^T,\,\,\, \check{v}_2=(-1,1)^T, \end{aligned}$$

and the infinitesimal transition rates are

3.10 $$\begin{aligned} \begin{aligned}&{\check{Q}}_{{\check{x}},{\check{x}}+\check{v}_1}=f_1({\check{x}}) = \alpha (\text {N}_{\text {tot}}-(x_1+x_2)),\,\,\, {\check{Q}}_{{\check{x}},{\check{x}}+\check{v}_2}=f_2({\check{x}}) = \beta x_1. \end{aligned} \end{aligned}$$

We now determine a coclique level function and the associated coclique level structure for the projected Markov chain ${\check{X}}$. To this end, consider the partition of the edge set given by $\mathcal {E}_+=\{e_1,e_2\},\, \mathcal {E}_-=\emptyset$. According to Lemma 3.2, since $\mathcal {G}$ is weakly connected, and Assumption 3.1 is satisfied, the system of equations (3.4) admits either zero or one solution. In this case, solving the system of equations

$$\begin{aligned} b_1 =1,\, -b_1 + b_2 = 1 \end{aligned}$$

yields the unique solution $b =(b_1,b_2)^T= (1,2)^T$. Therefore, the function $L(x_1,x_2) = x_1 + 2x_2$ is a coclique level function for ${\check{X}}$. The corresponding coclique level structure is the (ordered) partition $\{\mathcal {L}_{\ell },\dots ,\mathcal {L}_u\}$ of the state space ${\check{\mathcal {X}}}$, where each level set is defined as $\mathcal {L}_z := \left\{ x \in {\check{\mathcal {X}}} : L(x) = z \right\} , z = \ell , \dots , u$, with $\ell = \min \{L(x) : x \in {\check{\mathcal {X}}}\}=0$ and $u = \max \{L(x) : x \in {\check{\mathcal {X}}}\}=2\text {N}_{\text {tot}}$.

It is important to note that this is only one possible coclique level structure for ${\check{X}}$. In the next section (Sect. 4), we develop theoretical tools to identify all possible coclique level structures for SCRNs satisfying Assumption 3.1.

## Main results

In Sects. 4.1–4.3, we develop theoretical tools for SCRNs satisfying Assumption 3.1 and whose associated graph $\mathcal {G}$ is weakly connected, and in Sect. 4.4, we relax the latter condition and consider the case where $\mathcal {G}$ does not need to be weakly connected. More precisely, in Sect. 4.1, we introduce a theorem to determine all of the possible coclique level functions associated with SCRNs satisfying Assumption 3.1 and with $\mathcal {G}$ weakly connected. We then derive theoretical tools to determine when a projected continuous time Markov chain associated with a SCRN admits a coclique level function by studying the structure of the graph $\mathcal {G}$ associated with the SCRN. Additionally, we develop an algorithm to find, under certain assumptions, all of the possible coclique level functions associated with these SCRNs (see Sect. 4.2). We derive analytical formulas for upper and lower bounds on MFPTs for SCRNs having this type of structure (see Sect. 4.3). In Sect. 4.4, we generalize these results to study SCRNs that satisfy Assumption 3.1 and whose associated graph $\mathcal {G}$ does not need to be weakly connected. We apply our results to several examples in Sect. 5.

### Existence and characterization of coclique level structures

In the first theorem in this section, we show that if (3.4) admits a solution, then there is a coclique level function for ${\check{X}}$. Moreover, all coclique level functions for ${\check{X}}$ can be obtained in this way.

#### Theorem 4.1

*Consider a SCRN satisfying Assumption* 3.1. *Assume that the associated graph* $\mathcal {G}$ *is weakly connected*. *The set of all coclique level functions for* ${\check{X}}$ *is the set of all functions of the form* (3.3), *where* $b = (b_1,\dots ,b_{d-1})^T \in \mathbb {Z}^{d-1}$ *is a solution of the system* (3.4) *for some partition* $\{ \mathcal {E}_+, \mathcal {E}_-\}$ *of the edge set of* $\mathcal {G}$ (*and one of* $\mathcal {E}_+$ *and* $\mathcal {E}_-$ *may be empty)*.

#### Proof

Let *L* be a coclique level function for ${\check{X}}$. Then, by Sect. 3, *L* has the form (3.3), where $b \in \mathbb {Z}^{d-1}$ satisfies (3.4) with $\mathcal {E}_+=\{e_k:L(\check{v}_k)=1\}$ and $\mathcal {E}_-=\{e_k:L(\check{v}_k)=-1\}$. Conversely, suppose that *b* is a solution of the system (3.4) for some partition $\{ \mathcal {E}_+, \mathcal {E}_-\}$ of the edge set of $\mathcal {G}$ (where $\mathcal {E}_+$ or $\mathcal {E}_-$ may be empty). Then, $b \in \mathbb {Z}^{d-1}$ by Lemma 3.2, and with $L(x)=b^T x$ for all $x \in \mathbb {Z}^{d-1}$, *L* satisfies (3.2). Thus, *L* is a coclique level function for ${\check{X}}$. $\square$

#### Remark 4.1

For a given partition $\{ \mathcal {E}_+, \mathcal {E}_-\}$ of the edge set of $\mathcal {G}$, if the system (3.4) has a solution, then by Lemma 3.2, it has a unique solution. Furthermore, if we switch the labels of the two subsets, i.e., $\mathcal {E}_+$ becomes $\mathcal {E}_-$ and vice versa, the function *L* is replaced by $-L$, and the sets in the partition $\{\mathcal {L}_{\ell }, \dots , \mathcal {L}_u \}$ remain the same but the order is reversed. Therefore, we may consider the coclique level structures associated with *L* and $-L$ to be the same.

**Application to Example **3.1: Consider the SCRN introduced in (3.8), with associated graph $\mathcal {G}$ shown in Fig. 1b. As previously mentioned, by applying Lemma 3.1, we know that this SCRN has a unique conservation vector $m = (1,1,1)^T$. This allows us to introduce a projected continuous time Markov chain ${\check{X}}=\{ (X_1(t),X_2(t))^T:\, t \ge 0\}$, which keeps track of $(n_{\textrm{W}},n_{\textrm{Y}})$ through time, with transition vectors and associated rates given in (3.9) and (3.10), respectively.

Given that Assumption 3.1 is satisfied and the associated graph $\mathcal {G}$ is weakly connected, we can use Theorem 4.1 to determine all of the coclique level structures for ${\check{X}}$. To this end, consider all of the possible partitions $\{ \mathcal {E}_+, \mathcal {E}_-\}$ of edges of $\mathcal {G}$ that could allow us to determine a coclique level structure. These partitions are the following:

4.1 $$\begin{aligned}&\mathcal {E}_+=\{e_1,e_2\},\mathcal {E}_-=\emptyset \,\,\,\,\textrm{and}\,\,\,\,\mathcal {E}_+=\{e_1\},\mathcal {E}_-=\{e_2\}. \end{aligned}$$

We did not consider the partition $\mathcal {E}_+=\emptyset , \mathcal {E}_-=\{e_1,e_2\}$ or the partition $\mathcal {E}_+=\{e_2\}, \mathcal {E}_-=\{e_1\}$ because, as explained in Remark 4.1, the associated functions *L* would be the opposite of the ones obtained for the partitions considered above and the resulting coclique level structures are considered to be the same. For each partition, the system of equations in (3.4) has a unique solution, these being $(b_1,b_2)^T=(1,2)^T$ and $(b_1,b_2)^T=(1,0)^T$, respectively. Then, by applying Theorem 4.1, we can conclude that the projected Markov chain ${\check{X}}$ has two coclique level structures, with associated coclique level functions $L(x_1,x_2)=x_1+2x_2$ and $L(x_1,x_2)=x_1$, respectively.

Now, we characterize when there exists a coclique level function for ${\check{X}}$, assuming Assumption 3.1 and the associated graph $\mathcal {G}$ is weakly connected (see Theorem 4.3). In the course of this, we show how to derive a simple coclique level function, when one exists, which exploits bipartite structure of $\mathcal {G}$. There can be other coclique level functions and we can use Theorem 4.1 above to identify all possible coclique level functions.

Assume the SCRN satisfies Assumption 3.1 and its associated graph $\mathcal {G}$ is weakly connected. Consider a weakly directed cycle *c* in $\mathcal {G}$, i.e., a directed subgraph of $\mathcal {G}$ whose underlying undirected graph is a cycle. We abbreviate weakly directed cycle as wd-cycle.^3^ Choose an orientation for the cycle. Then, define the vector $\vartheta _c \in \mathbb {Z}^{n}$ associated with the wd-cycle *c* such that for $k=1,\dots ,n$,

4.2 $$\begin{aligned} \vartheta _c(k)= {\left\{ \begin{array}{ll} +1 & \hbox {if }e_k \hbox { is part of the wd-cycle and }e_k \hbox { is in}\\ & \hbox {the direction of the orientation of the wd-cycle,}\\ -1 & \hbox {if }e_k \hbox { is part of the wd-cycle and } e_k \hbox { is in }\\ & \hbox {the opposite direction of the orientation of the wd-cycle,}\\ 0 & \hbox {if }e_k \hbox { is not part of the wd-cycle.} \end{array}\right. } \end{aligned}$$

#### Theorem 4.2

*Consider a SCRN satisfying Assumption* 3.1*and assume its associated graph* $\mathcal {G}$ *is weakly connected.* *Let* $\mathcal {C}$ *be the set of all wd-cycles in* $\mathcal {G}$. *For each* $c \in \mathcal {C}$, *we have* $\vartheta _c\in \ker (S)$. *Furthermore, given a partition* $\{\mathcal {E}_+,\mathcal {E}_-\}$ *of the edge set of* $\mathcal {G}$ *(where one of* $\mathcal {E}_+,\mathcal {E}_-$ *may be empty), define a vector* $w$ *such that* $w_k=+1$ *if* $e_k \in \mathcal {E}_+$ *and* $w_k=-1$ *if* $e_k \in \mathcal {E}_-$. *Then, if* $w^T\vartheta _c \ne 0$ *for some* $c \in \mathcal {C}$, *the system* (3.4) *does not admit a solution* $b \in \mathbb {Z}^{d-1}$.

#### Proof

The $i^{th}$ entry of the vector $S\vartheta _c$ represents the amount of species $\textrm{S}_i$ consumed/produced after all the reactions associated with the wd-cycle *c* are triggered, where the signs in $\vartheta _c$ ensure that the edges are followed in the direction of the chosen orientation. Since *c* is a wd-cycle and Assumption 3.1 is satisfied, $S\vartheta _c=0$ and then $\vartheta _c\in \ker (S)$.

Since $$\check{S}^T$$ is $$S^T$$ without the last column, we have that $$\text {range} $$$$(\check{S}^T) \subseteq \text {range}(S^T) = \ker (S)^\perp$$. Since $$\vartheta _c\in \ker (S)$$ for each $$c \in \mathcal {C}$$, we have that $$\text {span}\{\vartheta _c: c \in \mathcal {C} \} \subset \ker (S)$$. It follows that if $$w^T\vartheta _c \ne 0$$ for some $$c \in \mathcal {C}$$, then $$w \notin \ker (S)^{\perp } \supseteq \text {range}(\check{S}^T)$$, and thus (3.4) does not admit a solution. $$\square$$

#### Corollary 4.1

*Consider a SCRN satisfying Assumption* 3.1 a*nd assume its associated graph* $\mathcal {G}$ *is weakly connected. Suppose *$\{\mathcal {E}_+,\mathcal {E}_-\}$ *is a partition of the edge set of G (where one of* $\mathcal {E}_+$ *and* $\mathcal {E}_-$ *can be empty). If there are* $v_k,v_{k'} \in \mathcal {V}$ *such that* $v_k=-v_{k'}$ *and the edges associated with them, *$e_k$ *and*$e_{k'}$*, belong to the same subset *$\mathcal {E}_+$ *or* $\mathcal {E}_-$, *then (*3.4*) does not admit a solution* $b \in \mathbb {Z}^{d-1}$*, and so there is no coclique level structure for this partition.*

#### Proof

Suppose that $e_k,e_{k'} \in \mathcal {E}_+$ have the properties described. Consider the wd-cycle *c* given by $e_k$ and $e_{k'}$, with corresponding vector $\vartheta _c$. Then, $\vartheta _c(\ell )=0$ for $\ell \notin \{k,k'\}$ and, depending on the orientation of the cycle, $\vartheta _c(k)=\vartheta _c(k')= +1$ or $\vartheta _c(k)=\vartheta _c(k')= -1$. Then, since both the edges belong to $\mathcal {E}_+$, we have $w$, defined as in (3.7), to be such that $w(k)=w(k')=1$ and $w(\ell )=0$ for $\ell \notin \{k,k'\}$. This implies that $|w^T\vartheta _c| = 2 \ne 0$. Then, the result follows from Theorem 4.2. Similar reasoning can be used for the case where $e_k,e_{k'}\in \mathcal {E}_-$.$\square$

#### Remark 4.2

Based on the results of Corollary 4.1, given a partition $\{ \mathcal {E}_+, \mathcal {E}_-\}$, we can obtain a coclique level function only if edges $e_k$ and $e_{k'}$ associated with $v_k,v_{k'} \in \mathcal {V}$ such that $v_k=-v_{k'}$ belong to different members of the partition. If this property holds, then the equation (3.4) associated with $v_k$ is the opposite of the one associated with $v_{k'}$. This implies that the two equations are providing the same information. Thus, in order to solve system (3.4) and reduce the computational cost, we can just write the system for ${\widehat{\mathcal {V}}}$, in which ${\widehat{\mathcal {V}}} \subseteq \mathcal {V}$ is a maximal set of reaction vectors such that, for any $v_k,v_{k'} \in {\widehat{\mathcal {V}}}$, $v_k \ne - v_{k'}$.

Finally, we introduce a theorem demonstrating that for a SCRN satisfying Assumption 3.1 and having a weakly connected graph $\mathcal {G}$, there is a coclique level structure for $\check{X}$ if and only if $\mathcal {G}$ is bipartite.

#### Theorem 4.3

*Consider a SCRN satisfying Assumption* 3.1*and suppose the associated graph* $\mathcal {G}$ *is weakly connected.* *If the graph* $\mathcal {G}$ *is bipartite, then there exists a coclique level function for the projected continuous time Markov chain* ${\check{X}}$. *In this case, by relabeling vertices if necessary, there are two disjoint, non-empty cocliques of vertices in* $\mathcal {G}$, $B=\{1,\dots ,{\bar{i}}\}$ *and* $C=\{{\bar{i}}+1,\dots ,d\}$, *and then*

4.3 $$\begin{aligned} L({\check{x}})=L(x_1,\dots ,x_{{\bar{i}}},x_{{\bar{i}}+1},\dots ,x_{d-1})=x_1+\dots +x_{{\bar{i}}} \end{aligned}$$

*defines a coclique level function for* ${\check{X}}$. *If the graph* $\mathcal {G}$ *is not bipartite, there is no coclique level structure for* ${\check{X}}$.

#### Proof

We start by assuming the graph $\mathcal {G}$ is bipartite, and show there exists a coclique level function for the projected continuous time Markov chain ${\check{X}}$ as described in the theorem. Suppose $\mathcal {G}$ is bipartite. By relabeling vertices if necessary, there are sets *B* and *C* as described in the theorem. Let $\mathcal {E}_+= \{ e_k = (i,j) : v_k \in \mathcal {V}, i \in C \text { and } j \in B\}$ and $\mathcal {E}_-= \{ e_k = (i,j) : v_k \in \mathcal {V}, i \in B \text { and } j \in C\}$. Note that this $\{ \mathcal {E}_+, \mathcal {E}_-\}$ is a partition of the edge set of $\mathcal {G}$ where one of $\mathcal {E}_+$ or $\mathcal {E}_-$ could be empty. Let $b \in \mathbb {Z}^{d-1}$ be the vector where the first ${\bar{i}}$ entries are 1 and the remaining $d-1-{\bar{i}}$ entries are zero and define $L(x) = b^Tx$ for $x \in \mathbb {Z}^{d-1}$. Since $\mathcal {G}$ is bipartite and *B* and *C* are two cocliques, each reaction corresponds either to an edge from a vertex in *C* to a vertex in *B* or an edge from a vertex in *B* to a vertex in *C*. In particular, when a reaction $v_k \in \mathcal {V}$ is triggered, if $e_k \in \mathcal {E}_+$ then $L(\check{v}_k)=+1$ and if $e_k \in \mathcal {E}_-$ then $L(\check{v}_k)=-1$. Thus, *L* satisfies (3.2) and is a coclique level function for ${\check{X}}$.

Now suppose the graph $\mathcal {G}$ is not bipartite. Then $\mathcal {G}$ contains at least one odd wd-cycle, $c^{odd}$ (see Theorem 5.1 on page 27 of Wilson 1996). Consider the vector $\vartheta _{c^{odd}}$ associated with this cycle. By definition, $\vartheta _{c^{odd}}$ has all of its entries equal to zero except for an odd number of entries, associated with the edges in the wd-cycle $c^{odd}$, which take values in $\{-1,+1\}$. For any partition $\{\mathcal {E}_+,\mathcal {E}_-\}$ of the edge set of $\mathcal {G}$ (where one of $\mathcal {E}_+$ or $\mathcal {E}_-$ may be empty), and for $w$ as in (3.7), the product $w^T\vartheta _{c^{odd}}$ will result in a sum of an odd number of terms, each of which is $+1$ or $-1$, and thus the sum can never be equal to zero. By Theorem 4.2, this implies that the system (3.4) does not admit a solution. By Theorem 4.1, there cannot be a coclique level function for ${\check{X}}$. $\square$

**Application to Example** 3.1: Consider the SCRN introduced in (3.8), with associated graph $\mathcal {G}$ shown in Fig. 1b. Earlier, we showed how, by applying Theorem 4.1, one can determine all of the coclique level functions for ${\check{X}}$. On the other hand, by inspecting $\mathcal {G}$, it is also possible to note that the graph is bipartite, and thus we can apply Theorem 4.3 to directly identify one of its coclique level functions. Specifically, the two disjoint, non-empty cocliques of vertices in $\mathcal {G}$ are $B=\{1\}$ and $C=\{2, 3\}$, and then $L(x)=x_1$ defines a coclique level function for ${\check{X}}$.

### Algorithm to determine all coclique level functions associated with a SCRN satisfying Assumption 3.1 and whose associated graph $\mathcal {G}$ is weakly connected

**Fig. 2:**
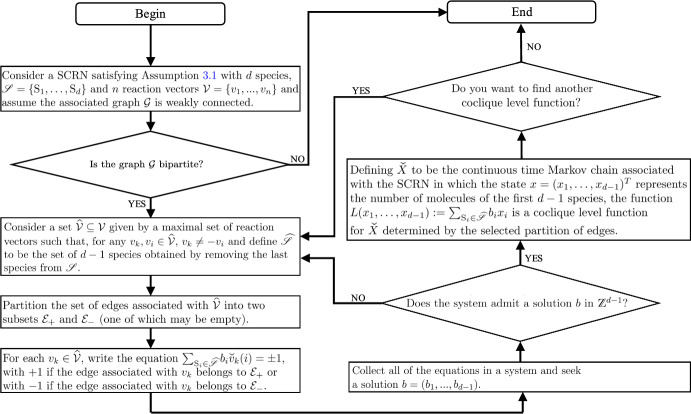
Key steps of the algorithm for identifying all coclique level functions for the projected continuous time Markov chain $\check{X}$ associated with a SCRN under Assumption 3.1 and assuming that $\mathcal {G}$ is weakly connected

Based on the results given in Theorems 4.1 and 4.3, given a SCRN satisfying Assumption 3.1 and assuming that $\mathcal {G}$ is weakly connected, we can obtain an algorithm that allows us to find all of the coclique level functions for the projected continuous time Markov chain ${\check{X}}$ associated with the SCRN. The steps of the algorithm are described in Fig. 2. The first step of the algorithm involves checking whether a graph is bipartite. The reader may refer to Section 3.4 in Kleinberg and Tardos (2005) for a discussion about a Breadth-First Search implementation to check whether a graph is bipartite, whose run time is linear in terms of the number of vertices in the graph (or, in our case, the number of species in the SCRN).

As a second step, the algorithm requires selecting a subset ${\widehat{\mathcal {V}}} \subseteq \mathcal {V}$ defined as a maximal set of reaction vectors such that $v_k \ne -v_i$ for any $v_k, v_i \in {\widehat{\mathcal {V}}}$. It is important to note that, once such a subset is identified, the specific ordering of the vectors in ${\widehat{\mathcal {V}}}$ does not affect the outcome of the algorithm. Moreover, the choice of ${\widehat{\mathcal {V}}}$ is uniquely determined up to the direction of the vectors and ensures that all directions allowed by the reactions are considered.

#### Remark 4.3

Note that the algorithm described here is for a specific ordering of the species associated with our specification of the SCRN.

### Using coclique level structure to bound MFPTs

As mentioned in the introduction, obtaining an explicit analytical expression for the mean first passage time (MFPT) for one-dimensional birth-death processes is relatively straightforward by using the formula (S.3) (see SI - Section S.3). However, it is typically very complicated to obtain an explicit expression for the MFPT for more complex Markov chains, especially in dimension greater than one. In this section, we will show how to obtain closed form formulas for upper and lower bounds for the MFPTs of continuous time Markov chains ${\check{X}}$ associated with SCRNs having a coclique level structure.

To this end, consider the projected continuous time Markov chain ${\check{X}}$ with finite state space ${\check{\mathcal {X}}} \subseteq \mathbb {Z}^{d-1}_{+}$, associated with a SCRN satisfying Assumption 3.1 and for which $\mathcal {G}$ is weakly connected (as defined in Sect. 3), and having infinitesimal generator ${\check{Q}}$. Suppose ${\check{X}}$ has a coclique level function $L: \mathbb {Z}^{d-1} \rightarrow \mathbb {Z}$, defined as in (3.2), with (ordered) partition $\{\mathcal {L}_{\ell },\mathcal {L}_{\ell +1}, \dots , \mathcal {L}_{u-1}, \mathcal {L}_u \}$ of $\check{\mathcal {X}}$, defined as in (3.5) and where $\ell < u$. Given such a coclique level structure for ${\check{X}}$, we will determine analytical expressions for upper and lower bounds for both the MFPT for ${\check{X}}$ from $\mathcal {L}_{\ell }$ to $\mathcal {L}_{u}$ and the MFPT for ${\check{X}}$ from $\mathcal {L}_{u}$ to $\mathcal {L}_{\ell }$. We first focus on the MFPT from $\mathcal {L}_{\ell }$ to $\mathcal {L}_{u}$.

Let

$$\begin{aligned} G_{+} := \{k:L(\check{v}_k) =1\} \quad \text { and } \quad G_{-} := \{k:L(\check{v}_k) =-1\}. \end{aligned}$$

For $\ell < u$, $\ell \le z \le u$ and ${\check{x}} \in \mathcal {L}_{z}$, define the “rate of increase” $\lambda _z({\check{x}})$ and the “rate of decrease” $\gamma _z({\check{x}})$ as follows:

$$\begin{aligned} \lambda _z({\check{x}})=\sum _{k \in G_{+}}{\check{Q}}_{{\check{x}},{\check{x}}+\check{v}_k},\,\,\,\,\gamma _z({\check{x}})=\sum _{k \in G_{-}}{\check{Q}}_{{\check{x}},{\check{x}}+\check{v}_k}. \end{aligned}$$

Then, for each $z\in \{\ell ,\ell +1,\dots ,u-1,u\}$, let

4.4 $$\begin{aligned} \begin{aligned}&\lambda ^M_z=\max _{{\check{x}}\in \mathcal {L}_z}\lambda _z({\check{x}}),\,\,\,\,\lambda ^m_z=\min _{{\check{x}}\in \mathcal {L}_z}\lambda _z({\check{x}}),\,\,\,\,\gamma ^M_z=\max _{{\check{x}}\in \mathcal {L}_z}\gamma _z({\check{x}}),\,\,\,\,\gamma ^m_z=\min _{{\check{x}}\in \mathcal {L}_z}\gamma _z({\check{x}}). \end{aligned} \end{aligned}$$

We will define continuous time Markov chains ${\breve{X}}$ and $\overset{\frown}{\mathit{X}}$ with the same state space as ${\check{X}}$. For $\ell \le z \le u$ and ${\check{x}} \in \mathcal {L}_{z}$, let

4.5 $$\begin{aligned} G_{+} ({\check{x}}) = \{k \in G_{+}: {\check{x}}+\check{v}_k \in {\check{\mathcal {X}}} \} \quad \text { and } \quad G_{-} ({\check{x}}) = \{k \in G_{-}: {\check{x}}+\check{v}_k \in {\check{\mathcal {X}}} \}. \end{aligned}$$

We assume that $G_{+} ({\check{x}}) : {\check{x}} \in {\check{\mathcal {X}}}{\setminus } \mathcal {L}_u$ are all non-empty sets when $G_+$ is non-empty, and $G_{-} ({\check{x}}): {\check{x}} \in {\check{\mathcal {X}}}\setminus \mathcal {L}_\ell$ are all non-empty sets when $G_-$ is non-empty. Note that $G_{+} ({\check{x}})$ is empty when ${\check{x}} \in \mathcal {L}_u$ and $G_{-} ({\check{x}})$ is empty when ${\check{x}} \in \mathcal {L}_\ell$. Then, we define the infinitesimal generator ${\breve{Q}}$ for $\breve{X}$ such that the only positive entries of ${\breve{Q}}$ are given by

$$\begin{aligned} {\breve{Q}}_{{\check{x}},{\check{x}}+\check{v}_k} = \frac{\lambda ^M_z}{|G_{+} ({\check{x}})|} \text { for } k \in G_{+} ({\check{x}}) \quad \text { and } \quad {\breve{Q}}_{{\check{x}},{\check{x}}+\check{v}_k} = \frac{\gamma ^m_z}{|G_{-}({\check{x}})|} \text { for } k \in G_{-} ({\check{x}}), \end{aligned}$$

for $\ell \le z \le u$ and ${\check{x}} \in \mathcal {L}_{z}$. Similarly, we define the infinitesimal generator $\overset{\frown}{\mathit{Q}}$ for $\overset{\frown}{\mathit{X}}$ such that the only positive entries of $\overset{\frown}{\mathit{Q}}$ are given byfor $\ell \le z \le u$ and ${\check{x}} \in \mathcal {L}_{z}$.

Then, by the comparison theorems, i.e., Theorems 3.3 and 3.4 in Campos et al. (2023) (see SI - Section S.4), using the matrix *A* equal to $b^T$ (associated with the coclique level function *L*), we have that for ${\check{x}}, \breve{x}$ and $\overset{\frown}{\mathit{x}}$ in $\mathcal {L}_{\ell }$, we can realize ${\check{X}}$ and $\breve{X}$ with infinitesimal generators ${\check{Q}}$ and ${\breve{Q}}$ (resp. ${\check{X}}$ and $\overset{\frown}{\mathit{X}}$ with infinitesimal generators ${\check{Q}}$ and $\overset{\frown}{\mathit{Q}}$) on the same probability space such that $L({\check{X}}) \le L(\breve{X})$, ${\check{X}}(0) = {\check{x}}$, $\breve{X}(0) = \breve{x}$ a.s. (resp. $L({\overset{\frown}{\mathit{X}}}) \le L({\check{X}})$,  $\overset{\frown}{\mathit{X}}(0) =\overset{\frown}{\mathit{X}}$, ${\check{X}}(0) = {\check{x}}$  a.s.). Then, for

$$\check{\tau }_{u} = \inf \{ t \ge 0:\, {\check{X}}(t) \in \mathcal {L}_u \}= \inf \{ t \ge 0:\, L({\check{X}}(t)) = u \},$$

$$\breve{\tau }_{u} = \inf \{ t \ge 0:\, \breve{X}(t) \in \mathcal {L}_u \}= \inf \{ t \ge 0:\, L(\breve{X}(t)) = u \},$$

and

$$\begin{aligned}\overset{\frown}{\tau }_{u}&=\inf \{ t \ge 0:\, \overset{\frown}{\mathit{X}}(t) \in \mathcal {L}_u \}\\ &= \inf \{ t \ge 0:\, L( \overset{\frown}{\mathit{X}}(t)) = u \},\end{aligned}$$

we have4.6By the choice of the rates for $\breve{X}$ and $\overset{\frown}{\mathit{X}}$, we have that $L(\breve{X})$ and $L(\overset{\frown}{\mathit{X}})$ are continuous time Markov chains. In fact, they are simple birth-death processes. It follows that the upper and lower bounds in (4.6) can be explicitly evaluated. For the lower bound, suppose that $\lambda ^M_z$ is positive for $\ell \le z \le u-1$. We replace $\lambda _i$ with $\lambda _z^M$ and $\gamma _i$ with $\gamma _z^m$, respectively, in formula (S.8), to obtain

4.7 $$\begin{aligned} \mathbb {E}_{\breve{x}}[\breve{\tau }_{u}]=\breve{h}_{\ell ,u}=\frac{1}{\lambda ^M_{u-1}} + \sum _{i=\ell }^{u-2} \frac{1}{\lambda ^M_{i}} \left( 1 + \sum _{j=i+1}^{u-1} \frac{\gamma ^m_{i+1}\dots \gamma ^m_{j}}{\lambda ^M_{i+1}\dots \lambda ^M_{j}} \right) . \end{aligned}$$

For the upper bound, suppose that $\lambda ^m_z$ is positive for $\ell \le z \le u-1$. We replace $\lambda _i$ with $\lambda _z^m$ and $\gamma _i$ with $\gamma _z^M$, respectively, in formula (S.8), to obtain4.8With a similar procedure, we can obtain lower and upper bounds for the MFPT for ${\check{X}}$ from $\mathcal {L}_{u}$ to $\mathcal {L}_{\ell }$. In particular, for $\check{\tau }_{\ell } = \inf \{ t \ge 0:\, {\check{X}}(t) \in \mathcal {L}_{\ell } \} = \inf \{ t \ge 0:\, L({\check{X}}(t)) = \ell \}$ and ${\check{x}}\in \mathcal {L}_u$, we have4.9in which4.104.11provided that, for (4.10), $\gamma ^M_z$ is positive for $\ell + 1 \le z \le u$, and for (4.11), $\gamma ^m_z$ is positive for $\ell + 1 \le z \le u$.

**Application to Example** 3.1: Consider the SCRN introduced in (3.8), with associated graph $$\mathcal {G}$$ shown in Fig. 1b. We seek lower and upper bounds for the MFPT from $$n_{\textrm{Z}}=\text {N}_{\text {tot}}$$ to $$n_{\textrm{Y}}=\text {N}_{\text {tot}}$$. To this end, let us consider the coclique level function $$L(x_1,x_2)=x_1+2x_2$$ previously identified. The coclique level structure associated with it can be written as $$\mathcal {L}_{\ell }, \dots , \mathcal {L}_u$$, with $$\mathcal {L}_z:=\{x\in {\check{\mathcal {X}}}:\, L(x_1,x_2)=x_1 $$$$ + 2x_2=z\}$$ for $$z=\ell ,\dots ,u$$, and $$\ell =0$$, $$u=2\text {N}_{\text {tot}}$$. This coclique level structure is such that $$(0,0)^T$$ (i.e., $$n_{\textrm{Z}}=\text {N}_{\text {tot}}$$) is the only state belonging to $$\mathcal {L}_\ell$$ and $$(0,\text {N}_{\text {tot}})^T$$ (i.e., $$n_{\textrm{Y}}=\text {N}_{\text {tot}}$$) is the only state belonging to $$\mathcal {L}_u$$ (Fig. 1c). This feature is critical in order to determine good lower and upper bounds for the MFPT from $$n_{\textrm{Z}}=\text {N}_{\text {tot}}$$ to $$n_{\textrm{Y}}=\text {N}_{\text {tot}}$$.

Now,

4.12 $$\begin{aligned} G_{+}=\{1,2\}\,\,\,\textrm{and}\,\,\,G_{-}=\emptyset . \end{aligned}$$

The rate of increase $\lambda _z({\check{x}})$ and the rate of decrease $\gamma _z({\check{x}})$ can be written as

4.13 $$\begin{aligned} \lambda _z({\check{x}})=f_1({\check{x}})+f_2({\check{x}})\,\,\,\textrm{and}\,\,\,\gamma _z({\check{x}})=0, \end{aligned}$$

with $f_1({\check{x}})$, $f_2({\check{x}})$ defined in (3.10).

The two continuous time Markov chains, $\breve{X}$ and $\overset{\frown}{\mathit{X}}$, are defined on the same state space as ${\check{X}}$, with infinitesimal generators ${\breve{Q}}$ and $\overset{\frown}{\mathit{Q}}$, respectively, such that, for $z\in \{\ell , \ell +1 ,\dots , u-1,u\}$ and ${\check{x}}\in \mathcal {L}_z$, ${\breve{Q}}_{{\check{x}},{\check{x}}+\check{v}_k} = \frac{\lambda ^M_z}{|G_{+} ({\check{x}})|}$ for $k \in G_{+} ({\check{x}})$,  , with $\lambda ^{M}_z=\max _{{\check{x}}\in \mathcal {L}_z}\lambda _z({\check{x}})$ and $\lambda ^{m}_z=\min _{{\check{x}}\in \mathcal {L}_z}\lambda _z({\check{x}})$, as defined in (4.4), and $G_{+} ({\check{x}})$ defined as in (4.5) where $G_{+}$ is given in (4.12). Note that in this example, $G_{-} =\emptyset$.

Then, as described in Section 4.3, we obtain analytical expressions for lower and upper bounds for the MFPT for ${\check{X}}$ from $n_{\textrm{Z}}=\text {N}_{\text {tot}}$ to $n_{\textrm{Y}}=\text {N}_{\text {tot}}$ asrespectively. Given that $\lambda ^{M}_z=\max _{{\check{x}}\in \mathcal {L}_z} \left( \alpha (\text {N}_{\text {tot}}-(x_1+x_2)) + \beta x_1\right)$ and $\lambda ^{m}_z=\min _{{\check{x}}\in \mathcal {L}_z} \left( \alpha (\text {N}_{\text {tot}}-(x_1+x_2)) + \beta x_1\right)$, where the functions being maximized and minimized are increasing in $\alpha$, respectively $\beta$, we see that the lower and upper bounds $\breve{h}_{\ell ,u}$ and  decrease as either of the rate constants $\alpha$ or $\beta$ increases. For this example, due to the simplicity of the SCRN considered, it is also possible to derive a compact analytical expression for the MFPT. Each of the $\text {N}_{\text {tot}}$ molecules acts independently and must undergo one ${\textrm{Z} \rightarrow \textrm{W}}$ transition and one ${ \textrm{W} \rightarrow  \textrm{Y}}$ transition to be converted from Z to Y. The time for one molecule of Z to be converted to Y is the sum of two independent exponential random variables with parameters $\alpha$ and $\beta$, respectively, which has a hypoexponential distribution with distribution function

$$\begin{aligned} F(x)= & (F_1 * F_2) (x)\\= & \int _0^x (1-e^{-\alpha (x-y)}) \beta e^{-\beta y} dy = \int _0^x (1-e^{-\beta (x-y)}) \alpha e^{-\alpha y} dy, \end{aligned}$$

for $x>0$ where $F_1$ and $F_2$ are distribution functions for exponential random variables with parameters $\alpha$ and $\beta$, respectively. The MFPT we desire is the mean of the maximum of $\mathrm {N_{tot}}$ such independent hypoexponential random variables, which is given by $\int _{0}^{\infty }(1-F(x)^{\mathrm {N_{tot}}})dx$. Observe that for fixed $\beta >0$ and $0<y<x$, $1-e^{-\alpha (x-y)}$ is increasing with $\alpha$, and then *F*(*x*) is increasing with $\alpha$. Thus, the MFPT decreases with increasing $\alpha$. Similarly, for fixed $\alpha >0$, we have that the MFPT decreases with increasing $\beta$.

Overall, this formulation explicitly gives the dependence of the MFPT on the rate constants $\alpha$ and $\beta$, and is fully consistent with the monotonic behavior that was observed through our study of its bounds.

### Generalization to the non-weakly-connected case

In this section, we will consider SCRNs satisfying Assumption 3.1 and whose associated graphs do not necessarily have to be weakly connected. In this case, the graph $\mathcal {G}$ associated with a SCRN can be decomposed into finitely many weakly connected components, and we use $p$ to denote the number of weakly connected components in $\mathcal {G}$. For each $q= 1, \dots , p$, we use $\mathcal {G}^q$ to denote the $q^{th}$ weakly connected component of $\mathcal {G}$ and use $d_q$ to denote the number of vertices in $\mathcal {G}^q$.

#### Example 4.1

Consider a SCRN with four species ($\textrm{S}_1$, $\textrm{S}_2$, $\textrm{S}_3$, $\textrm{S}_4$) and the following three reactions under mass-action kinetics:

4.14 $$\begin{aligned} {\textcircled {\small 1}}\,\textrm{S}_1{\mathop {\longrightarrow }\limits ^{\kappa _1}}\textrm{S}_2, \quad {\textcircled {\small 2}}\,\textrm{S}_2{\mathop {\longrightarrow }\limits ^{\kappa _2}}\textrm{S}_1, \quad {\textcircled {\small 3}}\,\textrm{S}_3{\mathop {\longrightarrow }\limits ^{\kappa _3}}\textrm{S}_4, \end{aligned}$$

where the reaction rate constants $\kappa _1,\kappa _2,\kappa _3$ are positive. The associated graph $\mathcal {G}$ can be represented as follows:

$$\begin{aligned} { {\small (1)}}\,\textrm{S}_1\mathop{\rightleftharpoons}\limits_{e_2}^{e_1}\textrm{S}_2, \quad { {\small (2)}}\,\textrm{S}_3{\mathop {\longrightarrow }\limits ^{e _3}}\textrm{S}_4.\end{aligned}$$

In this case, $\mathcal {G}$ has two weakly connected components. The dynamics of $\{\textrm{S}_1, \textrm{S}_2\}$ evolve independently from those of $\{\textrm{S}_3, \textrm{S}_4\}$, and we can analyze the two subsystems independently. We will show, at the end of this section, how one can study the whole system by studying each subsystem independently.

**Fig. 3:**
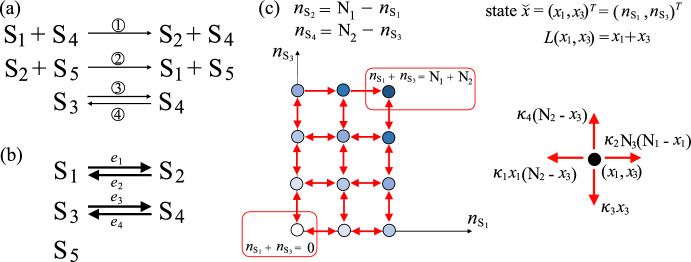
Example of a SCRN whose associated graph $\mathcal {G}$ is not weakly connected (Example 4.2). **a** Chemical reaction system diagram. The numbers on the arrows correspond to the reactions associated with the arrows as described in (4.15) in the main text. **b** Graph $\mathcal {G}$ associated with the chemical reaction system in panel (a). **c** State space and transitions of the projected continuous time Markov chain ${\check{X}}=\{(X_1(t),X_3(t))^T:\, t \ge 0\}$, which keeps track of $(n_{\mathrm {S_1}},n_{\mathrm {S_3}})$ through time. Here, we consider $N_1=2$, $N_2=3$ and we use dots to represent the states, and red double-ended (single-ended) arrows to represent transitions in both directions (in a single direction). Additionally, we use shades of blue to distinguish the level to which each state belongs. The function *L* associated with the coclique level structure is $L(x_1,x_3)=x_1+x_3$. On the right-hand side of the panel, we show the rates associated with the one-step transitions for the projected Markov chain ${\check{X}}$. Note that, given an initial condition, the quantity of species $\textrm{S}_5$ does not change over time and we denote this conserved quantity by $\mathrm {N_3}$

#### Example 4.2

For a reaction edge $e_k$ lying in some weakly connected component of $\mathcal {G}$, the infinitesimal rate $\Upsilon _k$ may depend on the quantities of some species where the graph vertices for those species may be in other weakly connected components of $\mathcal {G}$. For example, consider the following SCRN under mass-action kinetics:

4.15 $$\begin{aligned} \begin{aligned}&{\textcircled {\small 1}}\,{\textrm{S}_1 + \textrm{S}_4} {\mathop {\longrightarrow }\limits ^{\kappa _1}}{\textrm{S}_2 + \textrm{S}_4}, \quad {\textcircled {\small 3}}\,{\textrm{S}_3} {\mathop {\longrightarrow }\limits ^{\kappa _3}}{\textrm{S}_4}, \\&{\textcircled {\small 2}}\,{\textrm{S}_2 + \textrm{S}_5} {\mathop {\longrightarrow }\limits ^{\kappa _2}}{\textrm{S}_1 + \textrm{S}_5}, \quad {\textcircled {\small 4}}\,{\textrm{S}_4 } {\mathop {\longrightarrow }\limits ^{\kappa _4}}{\textrm{S}_3}, \end{aligned} \end{aligned}$$

where the reaction rate constants $\kappa _1, \kappa _2, \kappa _3, \kappa _4$ are positive. The diagram of this SCRN is shown in Fig. 3a and the associated graph $\mathcal {G}$ is shown in Fig. 3b. As a convention, we keep species $\textrm{S}_5$ even though its quantity is conserved over time, and the vertex representing $\textrm{S}_5$ in $\mathcal {G}$ is in its own weakly connected component. Here, $\mathcal {G}$ has three weakly connected components. The dynamics of $\{\textrm{S}_1, \textrm{S}_2\}$ do depend on the varying quantity of $\textrm{S}_4$, where $\textrm{S}_4$ is in a different weakly connected component from that of $\{\textrm{S}_1, \textrm{S}_2\}$. In this case, we cannot analyze the first two subsystems independently.

Now, for a SCRN satisfying Assumption 3.1 and whose associated graph $\mathcal {G}$ has $p$ weakly connected components, we shall and do relabel the species so that $j \in \{1,\dots ,d \}$ belongs to the $q^{th}$ weakly connected component of $\mathcal {G}$ if and only if $\sum _{q'=1}^{q-1} d_{q'} < j \le \sum _{q'=1}^{q} d_{q'}$ where a sum over an empty set is considered to be 0. With this relabeling, for a state $x \in \mathcal {X}$, $1 \le q\le p$ and $1 \le i \le d_q$, we shall use $x^q_i$ to denote the $\left( i+\sum _{q'=1}^{q-1} d_{q'} \right) ^{th}$ entry of *x*. In other words, we have

4.16 $$\begin{aligned} x= & (x^1, \dots , x^p)^T \in \mathbb {Z}^d_+ \text { where } \nonumber \\ x^q= & (x^q_1, \dots , x^q_{d_q})^T \in \mathbb {Z}^{d_q}_+ \text { for } q= 1, \dots , p. \end{aligned}$$

For $q= 1, \dots , p$, each vertex in $\mathcal {G}^q$ corresponds to a species and each edge in $\mathcal {G}^q$ corresponds to a reaction, and in this sense, there is a stoichiometric matrix $S^q$ associated with $\mathcal {G}^q$. Since there is no edge between different weakly connected components of $\mathcal {G}$, the stoichiometric matrix *S* associated with the SCRN has the form^4^

4.17 $$\begin{aligned} S = \begin{bmatrix} S^1 & 0 & 0 \\ 0 & \ddots & 0 \\ 0 & 0 & S^p\end{bmatrix}. \end{aligned}$$

By applying Lemma 3.1 to each weakly connected component of $\mathcal {G}$, we can show that the rank of the stoichiometric matrix *S* is $d-p$ (which also implies that the chemical reaction network associated with $\mathcal {G}$, in the manner described in Remark 3.1, has deficiency zero) and there are $p$ linearly independent conservation vectors for this SCRN. As a result, we shall consider a **projected continuous time Markov chain** ${\check{X}}=\{{\check{X}}(t):\, t \ge 0\}$ in which the state $\check{x} \in \check{\mathcal {X}} \subset \mathbb {Z}_+^{d-p}$ tracks, for each $q=1,\dots ,p$, the number of molecules of each species in $\mathcal {G}^q$, except for the last species in $\mathcal {G}^q$, that is,

4.18 $$\begin{aligned} \check{x} = (\check{x}^1, \dots , \check{x}^p)^T \text { where } \check{x}^q= (x^q_1, \dots , x^q_{d_q-1})^T \text { for } q= 1, \dots , p. \end{aligned}$$

Here, we slightly abuse notation, where ${\check{x}}^q$ is a zero-dimensional vector if $|\mathcal {G}^q| = 1$. Note that the choice to express $x^q_{d_q}$ as a function of $x^q_i: 1 \le i \le d_q-1$ is without loss of generality, since the species can always be relabeled so that a species chosen to be expressed as a function of the others is the last one. The process ${\check{X}}$ is a continuous time Markov chain^5^ defined on the finite state space

$$\begin{aligned} \check{\mathcal {X}}&:=\left\{\vphantom {\sum _{i=1}^{d_q-1}} \check{x} = ( x^q_i)_{1 \le i \le d_q- 1, 1 \le q\le p} \in \mathbb {Z}_{+}^{d-p}:  \right. \\ &\qquad \left. ( x^q_i)_{1 \le i \le d_q, 1 \le q\le p} \in \mathcal {X}\text { where } x^q_{d_q} = x^q_{\textrm{tot}} - \sum _{i=1}^{d_q-1}x^q_i \text { for } q= 1, \dots , p\right\} \\ &\, \subset \left\{ \check{x} = ( x^q_i)_{1 \le i \le d_q- 1, 1\le q\le p} \in \mathbb {Z}_{+}^{d-p}: x^q_1+\dots +x^q_{d_q-1} \right. \\ &\qquad\qquad\qquad\qquad\qquad\qquad\qquad\qquad\qquad\qquad\left.  \le x^q_{\textrm{tot}} \text { for } q= 1, \dots , p \vphantom {x^q_1+\dots +x^q_{d_q-1}}\right\} ,\end{aligned}$$

where $x^q_{\textrm{tot}}=\sum _{i=1}^{d_q}X^q_i(0)$ for each $q= 1, \dots , p$. We will assume that $|{\check{\mathcal {X}}}| > 1$, and the infinitesimal generator of ${\check{X}}$ will be denoted by $\check{Q}$.

A **coclique level function** for ${\check{X}}$ is a linear function $L: \mathbb {Z}^{d-p} \rightarrow \mathbb {Z}$ such that for each $k= 1,\dots ,n$,

4.19 $$\begin{aligned} L(\check{v}_k) \in \{-1,+1\}, \end{aligned}$$

where $\check{v}_k\in \mathbb {Z}^{d-p}$ is the vector obtained from $v_k$ by removing the last element in each of the weakly connected components in $\mathcal {G}$, in a similar manner to how we obtain (4.18) from (4.16). If such an *L* exists, it can be written as

4.20 $$\begin{aligned} L(x) = b^T x\,\, \textrm{for}\, x \in \mathbb {Z}^{d-p}\, \mathrm {and\, some}\, b \in \mathbb {Z}^{d-p}, \end{aligned}$$

where, upon partitioning the set of edges of the associated graph $\mathcal {G}$ into two disjoint subsets $\mathcal {E}_+=\{e_k:L(\check{v}_k)=1\}$ and $\mathcal {E}_-=\{e_k:L(\check{v}_k)=-1\}$ (where one of these may be empty), the vector $b=(b_1,\dots ,b_{d-p})^T$ solves the system of equations

4.21 $$\begin{aligned} \sum _{i=1}^{d-p} b_{i} \check{v}_k(i)= {\left\{ \begin{array}{ll} +1\,\,\,\text {if }\, e_k \in \mathcal {E}_+\\ -1\,\,\,\text {if }\, e_k \in \mathcal {E}_-\\ \end{array}\right. } \,\,\,\,\, \text {for } k=1,\dots ,n. \end{aligned}$$

For a coclique level function *L*, a **coclique level structure** for ${\check{X}}$ can be defined similar to that in the case where $\mathcal {G}$ is weakly connected.

Now, we are going to characterize all coclique level structures for ${\check{X}}$. In the following theorem, if the $q^{th}$ weakly connected component $\mathcal {G}^q$ of $\mathcal {G}$ has more than one vertex, i.e., $|\mathcal {G}^q| > 1$, then we will consider a SCRN that consists of all the species and reactions associated with this weakly connected component. If $|\mathcal {G}^q| = 1$, then ${\check{x}}^q$ is a zero-dimensional vector and we do not need to consider this weakly connected component when constructing coclique level functions as in (4.22).

#### Theorem 4.4

*Consider a SCRN satisfying Assumption *3.1*and *$|{\check{\mathcal {X}}}| > 1$*. A coclique level function for the projected continuous time Markov chain *$\check{X}$ *exists if and only if for every weakly connected component *$\mathcal {G}^q$ *of the associated graph *$\mathcal {G}$ *satisfying *$|\mathcal {G}^q|>1$ *where* $q\in \{1,\dots ,p\}$*, an associated coclique level function exists. The set of all coclique level functions for* ${\check{X}}$ *is the set of all functions of the form*

4.22 $$\begin{aligned} L(\check{x}) = L (\check{x}^1, \dots , \check{x}^p) = \sum _{\begin{array}{c} q=1,\dots ,p: \\ |\mathcal {G}^q|>1 \end{array}} L^q(\check{x}^q) \end{aligned}$$

*where *$L^q: \mathbb {Z}^{d_q-1} \rightarrow \mathbb {Z}$ *is a coclique level function associated with *$\mathcal {G}^q$ *for* $q=1,\dots ,p$ *and* $|\mathcal {G}^q| > 1$*. In particular, a coclique level function for *${\check{X}}$ *exists if and only if *$\mathcal {G}$ *is bipartite.*

The proof of Theorem 4.4 can be found in SI - Section S.2. For this, we can break down the problem to look at each weakly connected component separately, which is made possible by the block structure of the stoichiometric matrix shown in (4.17).

Suppose the projected continuous time Markov chain ${\check{X}}$ with finite state space ${\check{\mathcal {X}}} \subseteq \mathbb {Z}^{d-p}_{+}$ has a coclique level function $L: \mathbb {Z}^{d-p} \rightarrow \mathbb {Z}$, defined as in (4.19), with coclique level structure $\mathcal {L}_{\ell },\mathcal {L}_{\ell +1}, \dots , \mathcal {L}_{u-1}, \mathcal {L}_u$ defined as in (3.5)–(3.6) with $\ell < u$. Similar to the reasoning in Remark 4.1, for a coclique level function *L* as in (4.22), the sets $\mathcal {L}_{\ell }, \dots , \mathcal {L}_u$ in the coclique level structures associated with *L* and $-L$ are the same, while the orderings of those sets in the two coclique level structure partitions are opposite. As a convention, we consider the coclique level structures associated with *L* and $-L$ to be the same. Similar to the discussion in Sect. 4.3, we can determine analytical expressions for upper and lower bounds for both the MFPT for ${\check{X}}$ from $\mathcal {L}_{\ell }$ to $\mathcal {L}_{u}$ and the MFPT for ${\check{X}}$ from $\mathcal {L}_{u}$ to $\mathcal {L}_{\ell }$.

**Application to Example **4.1: In this example, $\mathcal {G}$ has two weakly connected components, and $m_1 = (1,1,0,0)^T$ and $m_2 = (0,0,1,1)^T$ are two linearly independent conservation vectors for this system. Therefore, we can consider a projected continuous time Markov chain ${\check{X}}=\{({X}_1(t), {X}_3(t))^T: t \ge 0\}$, which tracks the number of $\textrm{S}_1$ and $\textrm{S}_3$ through time. The state space for ${\check{X}}$ is $\{ (x_1,x_3)^T \in \mathbb {Z}^2_+: 0 \le x_1 \le \mathrm {N_1}, 0 \le x_3 \le \mathrm {N_2} \}$, where $\mathrm {N_1} = X_1(0) + X_2(0)$ and $\mathrm {N_2} = X_3(0) + X_4(0)$. We shall use Theorem 4.4 to identify all of the coclique level structures for ${\check{X}}$. For the first weakly connected component $\mathcal {G}^1$ of $\mathcal {G}$, the only possible useful partitions of edges of $\mathcal {G}^1$ are $\mathcal {E}_+^1=\{e_2\},\mathcal {E}_-^1=\{e_1\}$ and $\mathcal {E}_+^1=\{e_1\},\mathcal {E}_-^1=\{e_2\}$, by Remark 4.2 and the fact that reactions ${\textcircled {\small 1}}$ and ${\textcircled {\small 2}}$ are a pair of reversible reactions. One then can verify that $L^1 ({\check{x}}^1) = x_1^1 = x_1$ and $L^1 ({\check{x}}^1) = - x_1^1 = - x_1$ are the two coclique level functions associated with $\mathcal {G}^1$. Similarly, $L^2 ({\check{x}}^2) = x_1^2 = x_3$ and $L^2 ({\check{x}}^2) = - x_1^2 = - x_3$ are the only coclique level functions associated with $\mathcal {G}^2$. By Theorem 4.4, the coclique level functions for ${\check{X}}$ are $L({\check{x}})=x_1+x_3$, $L({\check{x}})=x_1-x_3$, $L({\check{x}})=-x_1+x_3$ and $L({\check{x}})=-x_1-x_3$, where the first two give the two distinct coclique level structures for ${\check{X}}$ by similar reasoning to that in Remark 4.1.

Because the dynamics of $\{\textrm{S}_1, \textrm{S}_2\}$ evolve independently from those of $\{\textrm{S}_3, \textrm{S}_4\}$, we can choose to analyze the two subsystems independently. For example, ${\check{X}}^1=\{X_1(t): t \ge 0\}$ is itself a continuous time Markov chain that tracks the number of $\textrm{S}_1$ and we have shown that $L^1 ({\check{x}}^1) = x_1^1 = x_1$ is a coclique level function associated with $\mathcal {G}^1$. Thus, we may study the MFPT from ${\check{X}}^1 = 0$ to ${\check{X}}^1 = \mathrm {N_1}$. However, it is not possible to study this MFPT using a coclique function for the whole system, because $L({\check{x}})=x_1$ is not a coclique level function for ${\check{X}}$. In conclusion, when the dynamics of each subsystem in the SCRN evolve independently, it is beneficial to apply our theory in Sects. 4.1–4.3 to each subsystem separately.

**Application to Example **4.2: In this example, $\mathcal {G}$ has three weakly connected components, and $m_1 = (1,1,0,0,0)^T$, $m_2 = (0,0,1,1,0)^T$ and $m_3=(0,0,0,0,1)^T$ are three linearly independent conservation vectors for this system. Therefore, we can consider a projected continuous time Markov chain ${\check{X}}=\{(X_1(t), X_3(t))^T: t \ge 0\}$, which tracks the number of $\textrm{S}_1$ and $\textrm{S}_3$ through time (Fig. 3c). The state space for ${\check{X}}$ is $\{ (x_1,x_3)^T \in \mathbb {Z}^2_+: 0 \le x_1 \le \mathrm {N_1}, 0 \le x_3 \le \mathrm {N_2} \}$, where $\mathrm {N_1} = X_1(0) + X_2(0)$ and $\mathrm {N_2} = X_3(0) + X_4(0)$. Note that, given an initial condition, the quantity of species $\textrm{S}_5$ does not change over time and we shall denote this conserved quantity by $\mathrm {N_3}$. Accordingly, the trivial dynamics of the system associated with the third weakly connected component of $\mathcal {G}$ is omitted in the projected chain ${\check{X}}$. Similar to the previous example, we can use Theorem 4.4 to identify all of the coclique level structures for ${\check{X}}$, which are the coclique level structures associated with the coclique level functions $L({\check{x}})=x_1+x_3$ and $L({\check{x}})=x_1-x_3$.

As an example, we can use the coclique level structure associated with the coclique level function $L({\check{x}})=x_1+x_3$ to bound the MFPT from ${\check{x}}=(x_1,x_3)^T=(\mathrm {N_1},\mathrm {N_2})^T$ to $(0,0)^T$ above and below by (4.10) and (4.11), respectively, where the parameters in these two expressions are given by $\ell =0$, $u= \mathrm {N_1}+\mathrm {N_2}$ and for $\ell \le z \le u$,

$$\begin{aligned} \lambda ^m_z= & \min _{\begin{array}{c} 0 \le x_1 \le \mathrm {N_1}, \\ 0 \le x_3 \le \mathrm {N_2},\\ x_1+x_3=z \end{array}} \left( \kappa _2 (\mathrm {N_1} - x_1) \mathrm {N_3} + \kappa _4 (\mathrm {N_2} - x_3) \right) , \\ \lambda ^M_z= & \max _{\begin{array}{c} 0 \le x_1 \le \mathrm {N_1}, \\ 0 \le x_3 \le \mathrm {N_2},\\ x_1+x_3=z \end{array}} \left( \kappa _2 (\mathrm {N_1} - x_1) \mathrm {N_3} + \kappa _4 (\mathrm {N_2} - x_3) \right) , \\ \gamma ^m_z= & \min _{\begin{array}{c} 0 \le x_1 \le \mathrm {N_1}, \\ 0 \le x_3 \le \mathrm {N_2},\\ x_1+x_3=z \end{array}} \left( \kappa _1 x_1 (\mathrm {N_2} - x_3) + \kappa _3 x_3 \right) , \quad \\ \gamma ^M_z= & \max _{\begin{array}{c} 0 \le x_1 \le \mathrm {N_1}, \\ 0 \le x_3 \le \mathrm {N_2},\\ x_1+x_3=z \end{array}} \left( \kappa _1 x_1 (\mathrm {N_2} - x_3) + \kappa _3 x_3 \right) , \end{aligned}$$

in which one can verify that $\gamma ^M_z \ge \gamma ^m_z > 0$ for each $1 \le z \le \mathrm {N_1}+\mathrm {N_2}$.

## Examples

In this section, we consider three examples and study their stochastic behavior, in terms of MFPT, by exploiting the theoretical tools developed in this paper. The examples come from biological areas of epigenetics, neurobiology and ecology (Bruno et al. 2022a; Milo et al. 2002; Brglez et al. 1989). All the continuous time Markov chains associated with the SCRNs considered in our examples have a finite state space.

### Chromatin modification circuit including only histone modifications

Epigenetic regulation is the modification of the DNA structure, due to chromatin modifications, that determines if a gene is active or repressed. Various chromatin modifications affect the structure of DNA. In this example, we will consider only histone modifications, while in the next one, we will study a more complex model including also DNA methylation. More precisely, in this example we analyze a well-established model for a histone modification circuit (Dodd et al. 2007; Bruno et al. 2022a, b), which involves three species: unmodified nucleosome, denoted by D; nucleosome modified with repressive histone modifications, denoted by $\mathrm {D^R}$; nucleosome modified with activating histone modifications, denoted by $\mathrm {D^A}$. In this model, each histone modification catalyzes its own establishment on unmodified nucleosomes and catalyzes the erasure of the opposite modification (Dodd et al. 2007; Bruno et al. 2022a).

**Fig. 4:**
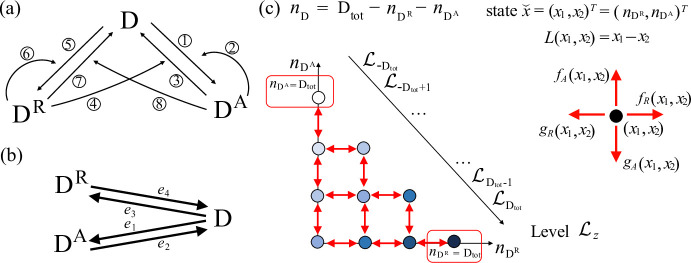
Chromatin modification circuit including only histone modifications: reaction diagram, graph $\mathcal {G}$ and associated Markov chain. **a** Chemical reaction system diagram. The numbers on the arrows correspond to the reactions associated with the arrows as described in (5.1) in the main text. **b** Graph $\mathcal {G}$ associated with the chemical reaction system in panel (a). **c** State space and transitions of the projected continuous time Markov chain ${\check{X}}=\{ (X_1(t),X_2(t))^T:\, t \ge 0\}$, which keeps track of $(n_{\mathrm {D^R}},n_{\mathrm {D^A}})$ through time. Here, we consider $\mathrm {D_{tot}}=3$ and we use dots to represent the states, and red double-ended arrows to represent transitions in both directions. Additionally, we use shades of blue to distinguish the level to which each state belongs. The function *L* associated with the coclique level structure is $L(x_1,x_2)=x_1-x_2$. The rates associated with the one-step transitions for the projected Markov chain ${\check{X}}$ are given in (5.2)

The quantity of each species is denoted by $n_{\textrm{D}}$, $n_{\mathrm {D^R}}$ and $n_{\mathrm {D^A}}$, respectively. Their sum remains constant, i.e., $n_{\textrm{D}}+n_{\mathrm {D^R}}+n_{\mathrm {D^A}}=\text {D}_{\text {tot}}$, where $\text {D}_{\text {tot}}$ denotes the total number of nucleosomes within the gene. Then, the chemical reaction system, whose diagram is shown in Fig. 4a, can be written as

5.1 $$\begin{aligned} \begin{aligned}&{\textcircled {\small 1}}\,{\textrm{D}} {\mathop { \xrightarrow{\hspace{30pt}}}\limits ^{k^A_{W0}+k^A_W}}{{\textrm{D}}^A },\,\,{\textcircled {\small 2}}\,{\textrm{D}} +{{\textrm{D}}^A} {\mathop {\longrightarrow }\limits ^{k^A_M }}{{\textrm{D}}^A + {\textrm{D}}^A},\,\,{\textcircled {\small 3}}\,{{\textrm{D}}^A} {\mathop {\xrightarrow{\hspace{30pt}} }\limits ^{\delta +{\bar{k}}^A_E}}{\textrm{D}},\\&{\textcircled {\small 4}}\,{{\textrm{D}}^A + {\textrm{D}}^{R}} {\mathop {\longrightarrow }\limits ^{k^A_E}}{{\textrm{D}} + {\textrm{D}}^{R}},\,\,{\textcircled {\small 5}}\,{\textrm{D}} {\mathop { \xrightarrow{\hspace{30pt}}}\limits ^{k^R_{W0}+k^R_{W}}}{{\textrm{D}}^R },\,\,\\&{\textcircled {\small 6}}\,{{\textrm{D}}+{\textrm{D}}^R} {\mathop {\longrightarrow }\limits ^{k^R_M}}{{\textrm{D}}^R + {\textrm{D}}^R},\\&{\textcircled {\small 7}}\,{{\textrm{D}}^R} {\mathop {\xrightarrow{\hspace{30pt}} }\limits ^{\delta +{\bar{k}}^R_E}}{\textrm{D}},\,\,{\textcircled {\small 8}}\,{{\textrm{D}}^R + {\textrm{D}}^{A}} {\mathop {\longrightarrow }\limits ^{k^R_E}}{{\textrm{D}} + {\textrm{D}}^{A}}, \end{aligned} \end{aligned}$$

in which $k^A_{W0},k^A_W,k^A_M,\delta , {\bar{k}}^A_E, k^A_E, k^R_{W0}, k^R_{W}, k^R_M, {\bar{k}}^R_E, k^R_E > 0$. The expression of the reaction rate constants is because we combined reactions sharing the same reactants and products. Now, denoting the reaction volume by *V*, let us introduce $\varepsilon := \frac{\delta + {\bar{k}}_E^A}{k_M^A(\text {D}_{\text {tot}}/V)} = \frac{\delta _A}{k_M^A(\text {D}_{\text {tot}}/V)}$, with $\delta _A:=\delta + {\bar{k}}_E^A$, and $\mu := \frac{k^R_E}{k^A_E}$. Furthermore, let us consider the constant ${\tilde{b}}$ such that $\mu {{\tilde{b}}} = \frac{\delta _{R}}{\delta _{A}}$, where $\delta _R:=\delta + {\bar{k}}_E^R$. Then, $\delta _R = \delta _A \mu {{\tilde{b}}} = \varepsilon \frac{k_M^A\text {D}_{\text {tot}}}{V} \mu {{\tilde{b}}}$.

Considering $x=(n_{\mathrm {D^R}},n_{\mathrm {D^A}},n_{\textrm{D}})^T$, the reaction vectors associated with (5.1) are $v_1=(0,1,-1)^T$, $v_2=(0,-1,1)^T$, $v_3=(1,0,-1)^T$, and $v_4=(-1,0,1)^T$. By examining them, it is possible to verify that Assumption 3.1 is satisfied. The graph $\mathcal {G}$ associated with the chemical reaction system (5.1) can then be represented as in Fig. 4b.

By inspecting $\mathcal {G}$, one can verify that the underlying undirected graph is connected and that $\mathcal {G}$ is bipartite. By Lemma 3.1, our SCRN has a unique conservation vector $m = (1,1,1)^T$ and then we can introduce a projected continuous time Markov chain ${\check{X}}=\{ (X_1(t),X_2(t))^T:\, t \ge 0\}$, which keeps track of $(n_{\mathrm {D^R}},n_{\mathrm {D^A}})$ through time. Since the total number of nucleosomes $\text {D}_{\text {tot}}$ is conserved, the state space is ${\check{\mathcal {X}}}= \{{\check{x}}=(x_1,x_2)^T \in \mathbb {Z}_+^2:\, x_1 + x_2 \le \text {D}_{\text {tot}}\}$. The potential one-step transitions for $\check{X}$ from $\check{x} \in {\check{\mathcal {X}}}$ are shown in Fig. 4c, where the associated transition vectors are $\check{v}_1=-\check{v}_2=(0,1)^T$ and $\check{v}_3=-\check{v}_4=(1,0)^T$, and the infinitesimal transition rates (in which we assume mass-action kinetics with the usual rate constant volume scaling) are

5.2 $$\begin{aligned} \begin{aligned}&{\check{Q}}_{{\check{x}},{\check{x}}+\check{v}_1}=f_A({\check{x}}) = (\text {D}_{\text {tot}}-(x_1+x_2))\left( k_{W0}^A+k_{W}^A + \frac{k_{M}^A}{V}x_2\right) ,\\&{\check{Q}}_{{\check{x}},{\check{x}}+\check{v}_2}=g_A({\check{x}}) = x_2\left( \varepsilon \frac{k_{M}^A}{V}\text {D}_{\text {tot}}+ x_1\frac{k^A_E}{V}\right) ,\\&{\check{Q}}_{{\check{x}},{\check{x}}+\check{v}_3}=f_R({\check{x}}) = (\text {D}_{\text {tot}}-(x_1+x_2))\left( k_{W0}^R+k_{W}^R + \frac{k_{M}^R}{V}x_1\right) ,\\&{\check{Q}}_{{\check{x}},{\check{x}}+\check{v}_4}=g_R({\check{x}}) = x_1\mu \left( \varepsilon \frac{k_{M}^A}{V}\text {D}_{\text {tot}}{{\tilde{b}}} + x_2\frac{k^A_E}{V}\right) . \end{aligned} \end{aligned}$$

Let us now focus on determining explicit analytical expressions for upper and lower bounds of MFPTs. To this end, let us apply Theorem 4.1 to determine the coclique level structures for ${\check{X}}$. We can apply the theorem because Assumption 3.1 is satisfied and the associated graph $\mathcal {G}$ is weakly connected.

Consider all of the possible partitions $\{ \mathcal {E}_+, \mathcal {E}_-\}$ of edges of $\mathcal {G}$ that could allow us to determine a coclique level structure. These partitions are the following:

5.3 $$\begin{aligned}&\mathcal {E}_+=\{e_1,e_3\},\mathcal {E}_-=\{e_2,e_4\}\,\,\,\,\textrm{and}\,\,\,\,\mathcal {E}_+=\{e_2,e_3\},\mathcal {E}_-=\{e_1,e_4\}. \end{aligned}$$

We did not consider the partition $\mathcal {E}_+=\{e_2,e_4\},$ $\mathcal {E}_-=\{e_1,e_3\}$ or the partition $\mathcal {E}_+=\{e_1,e_4\},$ $\mathcal {E}_-=\{e_2,e_3\}$ because, as explained in Remark 4.1, the associated functions *L* would be the opposite of the ones obtained for the partitions considered above and the resulting coclique level structures are considered to be the same. Furthermore, we did not consider the partitions in which two edges in the same element of a partition are associated with two reaction vectors $v_k, v_{k'} \in \mathcal {V}$ such that $v_k = -v_{k'}$, because, as stated in Corollary 4.1, these partitions would not lead to a coclique level structure. For each partition, the system of equations in (3.4) has a unique solution, these being $(b_1,b_2)^T=(1,1)^T$ and $(b_1,b_2)^T=(1,-1)^T$, respectively. Then, by applying Theorem 4.1, we can conclude that the projected Markov chain ${\check{X}}$ has two coclique level structures associated with coclique level functions $L(x_1,x_2)=x_1+x_2$ and $L(x_1,x_2)=x_1-x_2$.

Let us consider the coclique level function $L(x_1,x_2)=x_1-x_2$. The coclique level structure associated with it can be written as $\mathcal {L}_{\ell }, \dots , \mathcal {L}_u$, with $\mathcal {L}_z:=\{x\in {\check{\mathcal {X}}}:\, L(x_1,x_2)=x_1 - x_2=z\}$ for $z=\ell ,\dots ,u$, with $\ell =-\mathrm {D_{tot}}$ and $u=\mathrm {D_{tot}}$. This coclique level structure is such that $a=(0,\mathrm {D_{tot}})^T$ is the only state belonging to $\mathcal {L}_\ell$ and $r=(\mathrm {D_{tot}},0)^T$ is the only state belonging to $\mathcal {L}_u$ (see Fig. 4c). As shown below, this feature of the coclique level structure is critical in order to determine lower and upper bounds for $h_{a,r}$, the MFPT from *a* to *r*, and $h_{r,a}$, the MFPT from *r* to *a*, and this is the reason why we consider the coclique level structure associated with the function $L(x_1,x_2)=x_1-x_2$ and not the one associated with the function $L(x_1,x_2)=x_1+x_2$.

Let us now determine the lower and upper bounds for the MFPT from *a* to *r*, and vice versa, following the approach described in the previous section. In particular, here we have

5.4 $$\begin{aligned} G_{+}=\{2,3\}\,\,\,\textrm{and}\,\,\,G_{-}=\{1,4\}, \end{aligned}$$

and the rate of increase $\lambda _z({\check{x}})$ and the rate of decrease $\gamma _z({\check{x}})$ can be written as

5.5 $$\begin{aligned} \lambda _z({\check{x}})=f_R({\check{x}})+g_A({\check{x}})\,\,\,\textrm{and}\,\,\,\gamma _z({\check{x}})=f_A({\check{x}})+g_R({\check{x}}), \end{aligned}$$

with $f_R({\check{x}})$, $g_A({\check{x}})$, $f_A({\check{x}})$, $g_R({\check{x}})$ defined in (5.2). The two continuous time Markov chains $\breve{X}$ and $\overset{\frown}{\mathit{X}}$, defined on the same state space as ${\check{X}}$ have infinitesimal generators ${\breve{Q}}$ and $\overset{\frown}{\mathit{Q}}$, respectively, such that, for $z\in \{\ell , \ell +1 ,\dots , u-1,u\}$ and ${\check{x}}\in \mathcal {L}_z$, ${\breve{Q}}_{{\check{x}},{\check{x}}+\check{v}_k} = \frac{\lambda ^M_z}{|G_{+} ({\check{x}})|}$ for $k \in G_{+} ({\check{x}})$, ${\breve{Q}}_{{\check{x}},{\check{x}}+\check{v}_k} = \frac{\gamma ^m_z}{|G_{-}({\check{x}})|}$ for $k \in G_{-} ({\check{x}})$, $\overset{\frown}{\mathit{Q}}_{{\check{x}},{\check{x}}+\check{v}_k} = \frac{\lambda ^m_z}{|G_{+}({\check{x}})|}$ for $k \in G_{+} ({\check{x}})$, and $\overset{\frown}{\mathit{Q}}_{{\check{x}},{\check{x}}+\check{v}_k} = \frac{\gamma ^M_z}{|G_{-}({\check{x}})|}$ for $k \in G_{-} ({\check{x}})$, with $\lambda ^{M}_z$, $\lambda ^{m}_z$, $\gamma ^{M}_z$, and $\gamma ^{m}_z$ defined as in (4.4), and non-empty $G_{+} ({\check{x}})$ and $G_{-} ({\check{x}})$ defined as in (4.5) where $G_{+}$ and $G_{-}$ are given in (5.4).

Then, as described in Sect. 4.3, we can compare the Markov chain ${\check{X}}$ with $\breve{X}$ and $\overset{\frown}{\mathit{X}}$, separately, to conclude that5.6where $\breve{h}_{\ell ,u}$,  ,  , and $\breve{h}_{u,\ell }$ can be written as in (4.7), (4.8), (4.10), and (4.11), respectively, with the quantities in (4.4) replaced by those in (5.5).

These analytical expressions allow us to study the effect of the parameter $\varepsilon$ on the MFPT from *a* to *r* (average time to memory loss of the active state) and the MFPT from *r* to *a* (average time to memory loss of the repressed state). Specifically, given that the only $O(\varepsilon )$ rates are $\gamma ^{M}_u=\gamma ^{m}_u=g_R(\mathrm {D_{tot}},0)$ and $\lambda ^{M}_{\ell }=\lambda ^{m}_{\ell }=g_A(0,\mathrm {D_{tot}})$, with the other rates being *O*(1), we can conclude that the upper bounds  , $\breve{h}_{u,\ell }$ and the lower bounds $\breve{h}_{\ell ,u}$,  in (5.6) are all $O(\varepsilon ^{-1})$. This implies that the average time to memory loss of both the repressed and active states are $O(\varepsilon ^{-1})$, and as $\varepsilon$ approaches 0, the average time to memory loss of both the repressed and active states tends to infinity.

### Full chromatin modification circuit

**Fig. 5:**
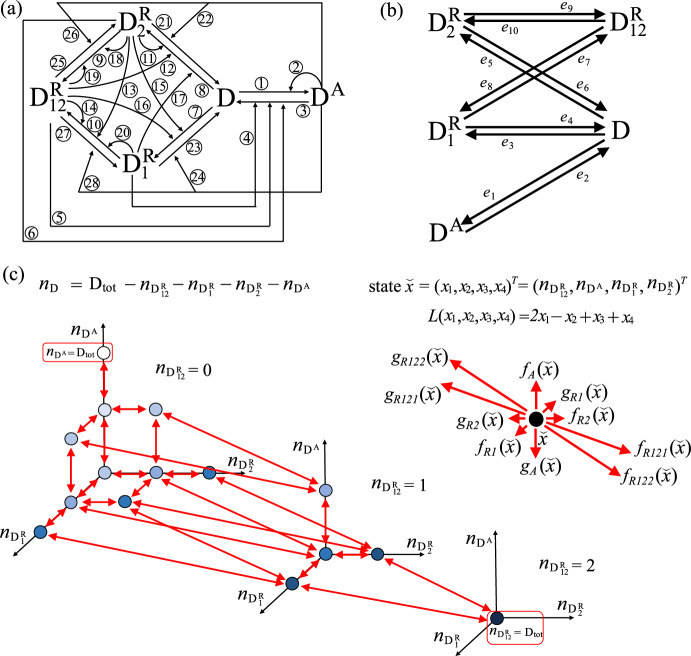
Full chromatin modification circuit: reaction diagram, graph $\mathcal {G}$ and associated Markov chain. **a** Chemical reaction system diagram. The numbers on the arrows correspond to the reactions associated with the arrows as described in (5.7) in the main text. **b** Graph $\mathcal {G}$ associated with the chemical reaction system in panel (a). **c** State space and transitions of the projected continuous time Markov chain ${\check{X}}=\{ (X_1(t),X_2(t),X_3(t),X_4(t))^T:\, t \ge 0\}$, which keeps track of $(n_{\mathrm {D^R_{12}}},n_{\mathrm {D^A}},n_{\mathrm {D^R_1}},n_{\mathrm {D^R_2}})$ through time. Here, we consider $\mathrm {D_{tot}}=2$ and we use dots to represent the states, and red double-ended arrows to represent transitions in both directions. Additionally, we use shades of blue to distinguish the level to which each state belongs. The function *L* associated with the coclique level structure is $L(x_1,x_2,x_3,x_4)=2x_1-x_2+x_3+x_4$. The rates associated with the one-step transitions for the projected Markov chain ${\check{X}}$ are given in (5.8)

In this example, we consider a chromatin modification circuit model that includes not only histone modifications, but also DNA methylation (Bruno et al. 2022a, 2023). The model involves five species: unmodified nucleosome, denoted by D; nucleosome with CpGme only, denoted by $\mathrm {D^R_1}$; nucleosome with H3K9me3 only, denoted by $\mathrm {D^R_2}$; nucleosome with both H3K9me3 and CpGme, denoted by $\mathrm {D^R_{12}}$; nucleosome with an activating histone modification, denoted by $\mathrm {D^A}$. In terms of molecular interactions, DNA methylation catalyzes the establishment of repressive histone modifications (and vice versa), while enhancing the erasure of activating marks (and vice versa) (Bruno et al. 2022a). The quantity of each species is denoted by $n_{\textrm{D}}$, $n_{\mathrm {D^A}}$, $n_{\mathrm {D^R_1}}$, $n_{\mathrm {D^R_2}}$, and $n_{\mathrm {D^R_{12}}}$, respectively. Their sum remains constant, i.e., $n_{\textrm{D}}+n_{\mathrm {D^A}}+n_{\mathrm {D^R_1}}+n_{\mathrm {D^R_2}}+n_{\mathrm {D^R_{12}}}=\text {D}_{\text {tot}}$. The chemical reaction system, whose diagram is shown in Fig. 5a, can be written as

5.7 $$\begin{aligned}&{\textcircled {\small 1}}\,\textrm{D} {\mathop { \xrightarrow{\hspace{30pt}} }\limits ^{k^A_{W0}+k^A_W}}{\textrm{D}^A },\,\,\,{\textcircled {\small 2}}\,{\textrm{D} + \mathrm {D^A}} {\mathop {\longrightarrow }\limits ^{k^A_M}}{\mathrm {D^A} + \mathrm {D^A} },\,\,\,{\textcircled {\small 3}}\,{\mathrm {D^A}} {\mathop {\xrightarrow{\hspace{30pt}} }\limits ^{\delta + {\bar{k}}^A_{E}}}{\textrm{D}},\nonumber \\&{\textcircled {\small 4}}\,{\mathrm {D^A} + \mathrm {D^R_1}} {\mathop {\longrightarrow }\limits ^{k^A_E}}{\textrm{D} + \mathrm {D^R_1} },\,\,\,{\textcircled {\small 5}}\,{\mathrm {D^A} + \mathrm {D^R_{12}}} {\mathop {\longrightarrow }\limits ^{2 k^A_E}}{\textrm{D} + \mathrm {D^R_{12}} },\,\,\,\nonumber \\ &{\textcircled {\small 6}}\,{\mathrm {D^A} + \mathrm {D^R_2}} {\mathop {\longrightarrow }\limits ^{k^A_E}}{\textrm{D} + \mathrm {D^R_2} },\nonumber \\&{\textcircled {\small 7}}\,{\textrm{D}} {\mathop { \xrightarrow{\hspace{30pt}} }\limits ^{k^1_{W0}+k^1_{W}}}{\mathrm {D^R_1} },\,\,\,{\textcircled {\small 8}}\,{\textrm{D}} {\mathop { \xrightarrow{\hspace{30pt}} }\limits ^{k^2_{W0}+k^2_{W}}}{\mathrm {D^R_2} },\,\,\,{\textcircled {\small 9}}\,{\mathrm {D^R_2}} {\mathop {\longrightarrow }\limits ^{k^1_{W0}}}{\mathrm {D^R_{12}} },\,\,\,{\textcircled {\small 10}}\,{\mathrm {D^R_1}} {\mathop {\longrightarrow }\limits ^{k^2_{W0}}}{\mathrm {D^R_{12}} },\nonumber \\&{\textcircled {\small 11}}\,{\textrm{D} + \mathrm {D^R_2}} {\mathop {\longrightarrow }\limits ^{k_M}}{\mathrm {D^R_2} + \mathrm {D^R_2} },\,\,\,{\textcircled {\small 12}}\;{\textrm{D} + \mathrm {D^R_{12}}} {\mathop { \xrightarrow{\hspace{30pt}} }\limits ^{k_M + {\bar{k}}_M}}{\mathrm {D^R_2} + \mathrm {D^R_{12}} },\nonumber \\&{\textcircled {\small 13}}\;{\mathrm {D^R_1} + \mathrm {D^R_2}} {\mathop {\longrightarrow }\limits ^{k_M}}{\mathrm {D^R_{12}} + \mathrm {D^R_2} },\;\;\;{\textcircled {\small 14}}\;{\mathrm {D^R_1} + \mathrm {D^R_{12}}} {\mathop { \xrightarrow{\hspace{30pt}} }\limits ^{k_M + {\bar{k}}_M}}{\mathrm {D^R_{12}} + \mathrm {D^R_{12}} },\nonumber \\&{\textcircled {\small 15}}\;{\textrm{D} + \mathrm {D^R_2}} {\mathop {\longrightarrow }\limits ^{k^{\prime }_M}}{\mathrm {D^R_1} + \mathrm {D^R_2} },\;\;\;{\textcircled {\small 16}}\;{\textrm{D} + \mathrm {D^R_{12}}} {\mathop {\longrightarrow }\limits ^{k^{\prime }_M}}{\mathrm {D^R_1} + \mathrm {D^R_{12}} },\;\;\;\nonumber \\ &{\textcircled {\small 17}}\;{\textrm{D} + \mathrm {D^R_1}} {\mathop {\longrightarrow }\limits ^{{\bar{k}}_M}}{\mathrm {D^R_2} + \mathrm {D^R_1} }\nonumber \\&{\textcircled {\small 18}}\;{\mathrm {D^R_2} + \mathrm {D^R_2}} {\mathop {\longrightarrow }\limits ^{k^{\prime }_M}}{\mathrm {D^R_{12}} + \mathrm {D^R_2} },\;\;\;{\textcircled {\small 19}}\;{\mathrm {D^R_2} + \mathrm {D^R_{12}}} {\mathop {\longrightarrow }\limits ^{k^{\prime }_M}}{\mathrm {D^R_{12}} + \mathrm {D^R_{12}} },\nonumber \\&{\textcircled {\small 20}}\;{\mathrm {D^R_1} + \mathrm {D^R_1}} {\mathop {\longrightarrow }\limits ^{{\bar{k}}_M}}{\mathrm {D^R_{12}} + \mathrm {D^R_1} },\;\;\; {\textcircled {\small 21}}\;{\mathrm {D^R_2}} {\mathop {\xrightarrow{\hspace{30pt}} }\limits ^{\delta +{\bar{k}}^R_{E}}}{\textrm{D} },\;\;\;{\textcircled {\small 22}}\;{\mathrm {D^R_2} + \mathrm {D^A}} {\mathop {\longrightarrow }\limits ^{k^R_E}}{\textrm{D} + \mathrm {D^A} },\nonumber \\&{\textcircled {\small 23}}\;{\mathrm {D^R_1}} {\mathop {\xrightarrow{\hspace{30pt}} }\limits ^{\delta ^{\prime }+k^{\prime }_{T}}}{\textrm{D}},\;\;\;{\textcircled {\small 24}}\;{\mathrm {D^R_1} + \mathrm {D^A}} {\mathop {\longrightarrow }\limits ^{k^{'*}_T}}{\textrm{D} + \mathrm {D^A} },\;\;\; {\textcircled {\small 25}}\;{\mathrm {D^R_{12}}} {\mathop {\xrightarrow{\hspace{30pt}} }\limits ^{\delta ^{\prime }+k^{\prime }_{T}}}{\mathrm {D^R_2} },\nonumber \\&{\textcircled {\small 26}}\;{\mathrm {D^R_{12}} + \mathrm {D^A}} {\mathop {\longrightarrow }\limits ^{k^{'*}_T}}{\mathrm {D^R_2} + \mathrm {D^A} },\;\;\;{\textcircled {\small 27}}\;{\mathrm {D^R_{12}}}{\mathop {\xrightarrow{\hspace{30pt}} }\limits ^{\delta +{\bar{k}}^R_{E}}}{\mathrm {D^R_1} },\;\;\;\nonumber \\&{\textcircled {\small 28}}\;{\mathrm {D^R_{12}} + \mathrm {D^A}} {\mathop {\longrightarrow }\limits ^{k^R_E}}{\mathrm {D^R_1} + \mathrm {D^A} }, \end{aligned}$$

in which $$k^A_{W0},k^A_W,k^A_M,\delta , {\bar{k}}^A_E,$$ $$k^A_E, k^1_{W0}, k^1_{W}, k^2_{W0}, k^2_{W},$$$$k'_M, {\bar{k}}_M, k_M, \delta ', k'_T, k^{'*}_T, {\bar{k}}^R_E,$$ $$  k^R_E > 0$$ and the expression of the reaction rate constants is because we combined reactions sharing the same reactants and products. As we did for Example 5.1, let us introduce parameters $$\varepsilon =\frac{\delta +\bar{k}^A_E}{\frac{k^A_M}{V}\text {D}_{\text {tot}}}$$ and $$\mu =\frac{k^R_E}{k^A_E}$$, with a constant $${{\tilde{b}}}$$ such that $$\frac{\delta +{\bar{k}}^R_E}{\delta + {\bar{k}}^A_E} = {{\tilde{b}}}\mu$$. Furthermore, since in this model we also have DNA methylation, we also introduce $$\mu '=\frac{k^{'*}_T}{k^A_E}$$ and a constant $$\beta$$ such that $$\frac{\delta ^{\prime }+k^{\prime }_T}{\delta + {\bar{k}}^A_E} = \beta \mu '$$. The parameter $$\mu '$$ quantifies the relative speed between the erasure rate of DNA methylation and the erasure rate of activating histone modifications.

Considering $x=(n_{\mathrm {D^R_{12}}},n_{\mathrm {D^A}},n_{\mathrm {D^R_1}},$ $n_{\mathrm {D^R_2}},n_{\textrm{D}})^T$, the reaction vectors associated with (5.7) are, $v_1=(0,1,0,0,-1)^T,\:v_2=(0,-1,0,0,1)^T,\:v_3=(0,0,1,0,-1)^T,$ $\:v_4=(0,0,-1,0,1)^T,\:v_5=(0,0,0,1,-1)^T,\:v_6=(0,0,0,-1,1)^T,\:v_7=(1,0,-1,0,0)^T,$ $\:v_8=(-1,0,1,0,0)^T,\:v_9=(1,0,0,-1,0)^T,\:$and$\:v_{10}=(-1,0,0,1,0)^T.\:$By examining them, one can verify that Assumption 3.1 is satisfied. The graph $\mathcal {G}$ associated with the chemical reaction system (5.7) can then be represented as in Fig. 5b. As done for Example 5.1, by inspecting $\mathcal {G}$, one can verify that the underlying undirected graph is connected and that $\mathcal {G}$ is bipartite. By Lemma 3.1, our SCRN has a unique conservation vector $m = (1,\ldots ,1)^T$ and we can introduce the projected continuous time Markov chain ${\check{X}}=\{ (X_1(t),X_2(t),X_3(t),X_4(t):t\geq 0\},$ which keeps track of $(n_{\mathrm {D^R_{12}}},n_{\mathrm {D^A}},$ $n_{\mathrm {D^R_1}},n_{\mathrm {D^R_2}})$ through time. Since the total number of nucleosomes $\text {D}_{\text {tot}}$ is conserved, the state space is ${\check{\mathcal {X}}}= \{{\check{x}}=(x_1,x_2,x_3,x_4)^T \in \mathbb {Z}_+^4$ $:\, x_1 + x_2 + x_3 + x_4\le \text {D}_{\text {tot}}\}$. The potential one-step transitions for $\check{X}$ from $\,\check x \in {\check{\mathcal {X}}}$ are shown in Fig. 5c, where the associated transition vectors are $\check{v}_1=-\check{v}_2=(0,1,0,0)^T$, $\check{v}_3=-\check{v}_4=(0,0,1,0)^T$, $\check{v}_5=-\check{v}_6=(0,0,0,1)^T$, $\check{v}_7=-\check{v}_8=(1,0,-1,0)^T$, and $\check{v}_9=-\check{v}_{10}=(1,0,0,-1)^T$, and the infinitesimal transition rates (in which we assume mass-action kinetics with the usual rate constant volume scaling) are

5.8 $$\begin{aligned}&{\check{Q}}_{{\check{x}},{\check{x}}+\check{v}_1}=f_A({\check{x}}) = (\text {D}_{\text {tot}}-(x_1+x_2+x_3+x_4))\left( k_{W0}^A+k_{W}^A + \frac{k_{M}^A}{V}x_2\right) ,\nonumber \\&{\check{Q}}_{{\check{x}},{\check{x}}+\check{v}_2}=g_A({\check{x}}) = x_2\left( \varepsilon \frac{k_{M}^A}{V}\text {D}_{\text {tot}}+ \frac{k^A_E}{V}(x_3+x_4+2x_1)\right) ,\nonumber \\&{\check{Q}}_{{\check{x}},{\check{x}}+\check{v}_3}=f_{R1}({\check{x}}) = (\text {D}_{\text {tot}}-(x_1+x_2+x_3+x_4))\left( k^1_{W0}+k^1_{W} + \frac{k^{\prime }_{M}}{V}(x_1+x_4)\right) ,\nonumber \\&{\check{Q}}_{{\check{x}},{\check{x}}+\check{v}_4}=g_{R1}({\check{x}}) = x_3\mu '\left( \varepsilon \frac{k_{M}^A}{V}\text {D}_{\text {tot}}\beta + x_2\frac{k^A_E}{V}\right) ,\nonumber \\&{\check{Q}}_{{\check{x}},{\check{x}}+\check{v}_5}=f_{R2}({\check{x}}) = (\text {D}_{\text {tot}}-(x_1+x_2+x_3+x_4))\nonumber \\ &\qquad \qquad\qquad\qquad\quad \cdot \left( k^2_{W0}+k^2_{W} + \frac{k_{M}}{V}(x_1+x_4) + \frac{{\bar{k}}_{M}}{V}(x_1+x_3)\right) ,\nonumber \\&Q_{{\check{x}},{\check{x}}+\check{v}_6}=g_{R2}({\check{x}}) = x_4\mu \left( \varepsilon \frac{k_{M}^A}{V}\text {D}_{\text {tot}}{{\tilde{b}}} + x_2\frac{k^A_E}{V}\right) ,\nonumber \\&{\check{Q}}_{{\check{x}},{\check{x}}+\check{v}_7}=f_{R121}({\check{x}}) = x_3\left( k^2_{W0}+ \frac{k_{M}}{V}(x_1+x_4) + \frac{{\bar{k}}_{M}}{V}\left( x_1+\frac{x_3-1}{2}\right) \right) ,\nonumber \\&{\check{Q}}_{{\check{x}},{\check{x}}+\check{v}_8}=g_{R121}({\check{x}}) = x_1\mu \left( \varepsilon \frac{k_{M}^A}{V}\text {D}_{\text {tot}}b + x_2\frac{k^A_E}{V}\right) , \nonumber \\&{\check{Q}}_{{\check{x}},{\check{x}}+\check{v}_9}=f_{R122}({\check{x}}) = x_4\left( k^1_{W0} + \frac{k^{\prime }_{M}}{V}\left( x_1+\frac{x_4-1}{2}\right) \right) ,\nonumber \\&{\check{Q}}_{{\check{x}},{\check{x}}+\check{v}_{10}}=g_{R122}({\check{x}}) = x_1\mu '\left( \varepsilon \frac{k_{M}^A}{V}\text {D}_{\text {tot}}\beta + x_2\frac{k^A_E}{V}\right) . \end{aligned}$$

A representation of the Markov chain graph for $\text {D}_{\text {tot}}=2$ is given in Fig. 5c.

We now focus on determining explicit analytical expressions for upper and lower bounds of the MFPT from the active state $a=(0,\mathrm {D_{tot}},0,0)^T$ to the repressed state $r=(\mathrm {D_{tot}},0,0,0)^T$, i.e., $h_{a,r}$, and vice versa, i.e., $h_{r,a}$, in order to understand how the parameters $\varepsilon$ and $\mu '$ affect them.

To calculate the MFPT upper and lower bounds, let us first apply Theorem 4.1 in order to determine the coclique level structures for ${\check{X}}$. We can apply the theorem because Assumption 3.1 is satisfied and the associated graph $\mathcal {G}$ is weakly connected. The rank of the stoichiometric matrix *S* is $4=d-1$.

Now, for this example, there are several possible partitions $\{ \mathcal {E}_+, \mathcal {E}_-\}$ of edges of $\mathcal {G}$ that could allow us to determine a coclique level structure. Let us consider the following one: $\mathcal {E}_+=\{e_2,e_3,e_5,e_7,e_9\},\mathcal {E}_-=\{e_1,e_4,e_6,e_8,e_{10}\}$. The reason for this choice is that it is the only one that, as we will see later, allows us to determine a coclique level structure in which the active state $a=(0,\mathrm {D_{tot}},0,0)^T$ and the repressed state $r=(\mathrm {D_{tot}},0,0,0)^T$ are the two extremum levels. For this partition, the system of equations in (3.4) admits a unique solution $(b_1,b_2,b_3,b_4)^T=(2,-1,1,1)^T$.

Then, by applying Theorem 4.1, we can conclude that the projected Markov chain ${\check{X}}$ has a coclique level structure associated with the coclique level function $L(x_1,x_2,x_3,x_4)=2x_1-x_2+x_3+x_4$.

This coclique level structure can be written as $\mathcal {L}_{\ell }, \dots , \mathcal {L}_u$, with $\mathcal {L}_z:=$ $\{x\in {\check{\mathcal {X}}}:\, L(x)=2x_1-x_2+x_3+x_4=z\}$ for $z=\ell ,\dots ,u$, with $\ell =-\mathrm {D_{tot}}$ and $u=2\mathrm {D_{tot}}$ (Fig. 5c). This coclique level structure is such that $a=(0,\mathrm {D_{tot}},0,0)^T$ is the only state belonging to $\mathcal {L}_\ell$ and $r=(\mathrm {D_{tot}},0,0,0)^T$ is the only state belonging to $\mathcal {L}_u$. Here,

5.9 $$\begin{aligned} G_{+}=\{2,3,5,7,9\}\;\;\;\textrm{and}\;\;\;G_{-}=\{1,4,6,8,10\}, \end{aligned}$$

and the rate of increase $\lambda _z({\check{x}})$ and the rate of decrease $\gamma _z({\check{x}})$ can then be written as

5.10 $$\begin{aligned} \begin{aligned}&\lambda _z({\check{x}})=f_{R121}({\check{x}})+f_{R122}({\check{x}})+g_A({\check{x}})+f_{R1}({\check{x}})+f_{R2}({\check{x}}),\\&\gamma _z({\check{x}})=f_A({\check{x}})+g_{R121}({\check{x}})+g_{R122}({\check{x}})+g_{R1}({\check{x}})+g_{R2}({\check{x}}), \end{aligned} \end{aligned}$$

respectively, with $f_{R121}({\check{x}})$, $f_{R122}({\check{x}})$, $g_A({\check{x}})$, $f_{R1}({\check{x}})$, $f_{R2}({\check{x}})$, $f_A({\check{x}})$, $g_{R121}({\check{x}})$, $g_{R122}({\check{x}})$, $g_{R1}({\check{x}})$, $g_{R2}({\check{x}})$ defined in (5.8).

The two continuous time Markov chains $\breve{X}$ and $\overset{\frown}{\mathit{X}}$ are defined on the same state space as ${\check{X}}$ and have infinitesimal generators ${\breve{Q}}$ and $\overset{\frown}{\mathit{Q}}$, respectively, such that, for $z\in \{\ell , \ell +1 ,\dots , u-1,u\}$ and ${\check{x}}\in \mathcal {L}_z$, ${\breve{Q}}_{{\check{x}},{\check{x}}+\check{v}_k} = \frac{\lambda ^M_z}{|G_{+} ({\check{x}})|}$ for $k \in G_{+} ({\check{x}})$, ${\breve{Q}}_{{\check{x}},{\check{x}}+\check{v}_k} = \frac{\gamma ^m_z}{|G_{-}({\check{x}})|}$ for $k \in G_{-} ({\check{x}})$, $\overset{\frown}{\mathit{Q}}_{{\check{x}},{\check{x}}+\check{v}_k} = \frac{\lambda ^m_z}{|G_{+}({\check{x}})|}$ for $k \in G_{+} ({\check{x}})$, and $\overset{\frown}{\mathit{Q}}_{{\check{x}},{\check{x}}+\check{v}_k} = \frac{\gamma ^M_z}{|G_{-}({\check{x}})|}$ for $k \in G_{-} ({\check{x}})$, with $\lambda ^{M}_z=\max _{{\check{x}}\in \mathcal {L}_z}\lambda _z({\check{x}})$, $\lambda ^{m}_z=\min _{{\check{x}}\in \mathcal {L}_z}\lambda _z({\check{x}}),$ $\gamma ^{M}_z=\max _{{\check{x}}\in \mathcal {L}_z}\gamma _z({\check{x}})$, and $\gamma ^{m}_z=\min _{{\check{x}}\in \mathcal {L}_z}\gamma _z({\check{x}})$, as defined in (4.4), and non-empty $G_{+} ({\check{x}})$ and $G_{-} ({\check{x}})$ defined as in (4.5) where $G_{+}$ and $G_{-}$ are given in (5.9).

Then, as described in Sect. 4.3, we can compare the Markov chain ${\check{X}}$ with $\breve{X}$ and $\overset{\frown}{\mathit{X}}$, separately, to obtain analytical expressions for lower and upper bounds, respectively, for the MFPT $h_{a,r}$ from the fully active state to the fully repressed state:
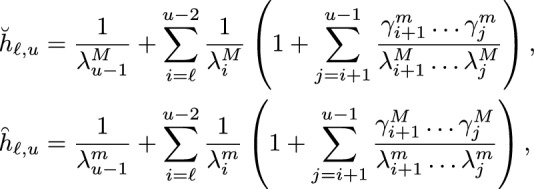
and expressions for lower and upper bounds for the MFPT $h_{r,a}$ from the fully repressed state to the fully active state:
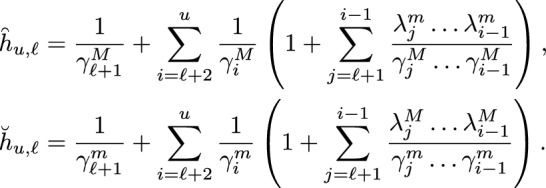
Given that the only $O(\varepsilon )$ rates are $\lambda ^{M}_{\ell }$, $\lambda ^{m}_{\ell }$, $\gamma ^{M}_u$, $\gamma ^{M}_{u-1}$, and $\gamma ^{m}_{z}:\text {D}_{\text {tot}}\le z \le u$, with the other rates being *O*(1), we can conclude that both $\breve{h}_{\ell ,u}$ and $\overset{\frown}{\mathit{h}}_{\ell ,u}$ are $O(\varepsilon ^{-1})$, and thus the MFPT from *a* to *r* (average time to memory loss of the active state) is $O(\varepsilon ^{-1})$. Furthermore, we have $\overset{\frown}{\mathit{h}}_{u, \ell}$ is $O(\varepsilon ^{-2})$ and $\breve{h}_{u,\ell }$ is $O(\varepsilon ^{-\text {D}_{\text {tot}}})$, and thus the MFPT from *r* to *a* (average time to memory loss of the repressed state) is at least $O(\varepsilon ^{-2})$. In cases like this, alternative approaches are needed to identify the precise scalings of MFPTs, such as the method we developed in our recent work (Bruno et al. 2024), which allows us to show that average time to memory loss of the repressed state is indeed $O(\varepsilon ^{-2})$. These results suggest that decreasing $\varepsilon$ extends the memory of both the active and repressed chromatin states, but with a more pronounced impact on the repressed state. This difference can be attributed to the cooperation of repressive chromatin marks, i.e., DNA methylation and repressive histone modifications, which introduces a structural bias in the chromatin modification circuit towards a repressed chromatin state.

Let us now determine the effect of $\mu '$, i.e., the parameter quantifying the relative speed between the DNA methylation erasure rate and the activating histone modification erasure rate, on the time to memory loss. Since the rates $g_{R1}({\check{x}})$ and $g_{R121}({\check{x}})$ are linear in $\mu '$ and are the only transition rates depending on $\mu '$ (see (5.8)), then, based on the definition in (5.10), $\gamma _z({\check{x}})$ increases for lower values of $\mu '$. This implies that increasing $\mu '$ leads to higher $\overset{\frown}{\mathit{h}}_{\ell ,u}$ and $\breve{h}_{\ell ,u}$ and lower $\breve{h}_{u,\ell }$ and $\overset{\frown}{\mathit{h}}_{u, \ell}$ . The opposite happens when $\mu '$ decreases.

### Bi-parallel network motif

**Fig. 6:**
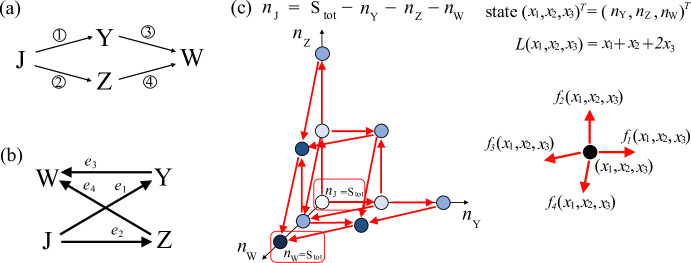
Bi-parallel network motif: reaction diagram, graph $\mathcal {G}$ and associated Markov chain. **a** Chemical reaction system diagram. The numbers on the arrows correspond to the reactions associated with the arrows as described in (5.1) in the main text. **b** Graph $\mathcal {G}$ associated with the chemical reaction system in panel (a). **c** State space and transitions for the projected continuous time Markov chain ${\check{X}}=\{ (X_1(t),X_2(t),X_3(t))^T:\, t \ge 0\}$, which keeps track of $(n_{\textrm{Y}},n_{\textrm{Z}},n_{\textrm{W}})$ through time. Here, we consider $\mathrm {S_{tot}}=2$ and we use dots to represent the states, and red double-ended arrows to represent transitions in both directions. Additionally, we use shades of blue to distinguish the coclique level to which each state belongs. The function $L(x_1,x_2,x_3)$ associated with the level structure is $L(x_1,x_2,x_3)=x_1+x_2+2x_3$. The rates associated with the one-step transitions for the projected Markov chain ${\check{X}}$ are given in (5.2)

In this example, we analyze a bi-parallel network motif, which is a typical building block found in complex networks (Milo et al. 2002), such as the neuronal connectivity network of the nematode Caenorhabditis elegans or food web networks (Milo et al. 2002; Brglez et al. 1989). The model includes four species, which are J, Y, Z, and W, and the quantity of each species is denoted by $n_{\textrm{J}}$, $n_{\textrm{Y}}$, $n_{\textrm{Z}}$, and $n_{\textrm{W}}$, respectively. Their sum remains constant, that is $n_{\textrm{J}}+n_{\textrm{Y}}+n_{\textrm{Z}}+n_{\textrm{W}}=\text {S}_{\text {tot}}$. Then, the chemical reaction system, whose diagram is shown in Fig. 6a, can be written as

5.11 $$\begin{aligned} \begin{aligned}&{\textcircled {\small 1}}\;\textrm{J} {\mathop {\longrightarrow }\limits ^{k_1}}\textrm{Y},\;\;{\textcircled {\small 2}}\;\textrm{J} {\mathop {\longrightarrow }\limits ^{k_2}}\textrm{Z},\;\;{\textcircled {\small 3}}\;\textrm{Y} {\mathop {\longrightarrow }\limits ^{k_3}}\textrm{W},\;\;{\textcircled {\small 4}}\;\textrm{Z} {\mathop {\longrightarrow }\limits ^{k_4}}\textrm{W}, \end{aligned} \end{aligned}$$

in which $$k_1,k_2,k_3,k_4 > 0$$.

Considering $x=(n_{\textrm{Y}},n_{\textrm{Z}},n_{\textrm{W}},n_{\textrm{J}})^T$, the reaction vectors associated with (5.11) are $v_1=(1,0,0,-1)^T$, $v_2=(0,1,0,-1)^T$, $v_3=(-1,0,1,0)^T$, and $v_4=(0,-1,1,0)^T$. By examining them, we see that Assumption 3.1 is satisfied. The graph $\mathcal {G}$ associated with the chemical reaction system (5.11) can be represented as in Fig. 6b. By inspecting $\mathcal {G}$, we see that the underlying undirected graph is connected and that $\mathcal {G}$ is bipartite. By Lemma 3.1, our SCRN has a unique conservation vector $m=(1,1,1,1)^T$ and then we can introduce a projected continuous time Markov chain ${\check{X}}=\{ (X_1(t),X_2(t),X_3(t))^T:\, t \ge 0\}$, which keeps track of $(n_{\textrm{Y}},n_{\textrm{Z}},n_{\textrm{W}})$ through time. Since $\text {S}_{\text {tot}}$ is conserved, the state space is ${\check{\mathcal {X}}}= \{{\check{x}}=(x_1,x_2,x_3)^T \in \mathbb {Z}_+^3:\, x_1 + x_2 + x_3 \le \text {S}_{\text {tot}}\}$. The potential one-step transitions for $\check{X}$ from $\check x \in {\check{\mathcal {X}}}$ are shown in Fig. 6c, where the associated transition vectors are $\check{v}_1=(1,0,0)^T$, $\check{v}_2=(0,1,0)^T$, $\check{v}_3=(-1,0,1)^T$, and $\check{v}_4=(0,-1,1)^T$, and the infinitesimal transition rates (in which we assume mass-action kinetics) are

5.12 $$\begin{aligned} \begin{aligned}&{\check{Q}}_{{\check{x}},{\check{x}}+\check{v}_1}=f_1({\check{x}}) = k_1(\text {S}_{\text {tot}}-(x_1+x_2+x_3)),\\&{\check{Q}}_{{\check{x}},{\check{x}}+\check{v}_2}=f_2({\check{x}}) = k_2(\text {S}_{\text {tot}}-(x_1+x_2+x_3)),\\&{\check{Q}}_{{\check{x}},{\check{x}}+\check{v}_3}=f_3({\check{x}}) = k_3 x_1,\;\; {\check{Q}}_{{\check{x}},{\check{x}}+\check{v}_4}=f_4({\check{x}}) = k_4x_2. \end{aligned} \end{aligned}$$

Let us now focus on determining explicit analytical expressions for upper and lower bounds of MFPTs. To this end, we apply Theorem 4.1 to determine the coclique level structures for ${\check{X}}$. We can apply the theorem because Assumption 3.1 is satisfied, and the associated graph $\mathcal {G}$ is weakly connected. The rank of the stoichiometric matrix *S* is $3=d-1$.

Now, consider all of the possible partitions $\{ \mathcal {E}_+, \mathcal {E}_-\}$ of edges of $\mathcal {G}$ that could allow us to determine a coclique level structure. These partitions are the following:

5.13 $$\begin{aligned}&\mathcal {E}_+=\{e_1,e_2,e_3,e_4\},\mathcal {E}_-=\emptyset ;\;\;\;\mathcal {E}_+=\{e_1\},\mathcal {E}_-=\{e_2,e_3,e_4\};\nonumber \\&\mathcal {E}_+=\{e_2\},\mathcal {E}_-=\{e_1,e_3,e_4\};\;\;\;\mathcal {E}_+=\{e_3\},\mathcal {E}_-=\{e_1,e_2,e_4\};\nonumber \\&\mathcal {E}_+=\{e_4\},\mathcal {E}_-=\{e_1,e_2,e_3\};\;\;\;\mathcal {E}_+=\{e_1,e_2\},\mathcal {E}_-=\{e_3,e_4\};\nonumber \\&\mathcal {E}_+=\{e_1,e_3\},\mathcal {E}_-=\{e_2,e_4\};\;\;\;\mathcal {E}_+=\{e_1,e_4\},\mathcal {E}_-=\{e_2,e_3\}. \end{aligned}$$

As was done for Example 5.1, we did not consider the partitions obtained by switching the labels of the subsets $\mathcal {E}_+$, $\mathcal {E}_-$ in the partitions listed above because, as explained in Remark 4.1, the associated functions *L* would be the opposite of the ones obtained for the partitions considered above. Therefore, the resulting coclique level structures may be considered to be the same.

For each partition, we can write the system of equations as in (3.4). The only systems that admit a solution $b \in \mathbb {Z}^3$ are the ones associated with the first, sixth, and eighth partitions. The solutions are $(b_1,b_2,b_3)^T=(1,1,2)^T$, $(b_1,b_2,b_3)^T=(1,1,0)^T$, and $(b_1,b_2,b_3)^T=(1,-1,0)^T$, respectively. Then, by applying Theorem 4.1, we can conclude that the projected Markov chain ${\check{X}}$ has three coclique level structures associated with the coclique level functions $L(x_1,x_2,x_3)=x_1+x_2+2x_3$, $L(x_1,x_2,x_3)=x_1+x_2$, and $L(x_1,x_2,x_3)=x_1-x_2$.

We consider the coclique level function $L(x_1,x_2,x_3)=x_1+x_2+2x_3$. The coclique level structure associated with it can be written as $\mathcal {L}_{\ell }, \dots , \mathcal {L}_u$, with $\mathcal {L}_z:=\{x\in {\check{\mathcal {X}}}:\, L(x_1,x_2,x_3)=x_1 + x_2+2x_3=z\}$ for $z=\ell ,\dots ,u$, with $\ell =0$ and $u=2\text {S}_{\text {tot}}$. This coclique level structure is such that $(0,0,0)^T$ (i.e., $n_{\textrm{J}}=\text {S}_{\text {tot}}$) is the only state belonging to $\mathcal {L}_\ell$ and $(0,0,\text {S}_{\text {tot}})^T$ (i.e., $n_{\textrm{W}}=\text {S}_{\text {tot}}$) is the only state belonging to $\mathcal {L}_u$ (Fig. 6c). This feature, as shown in the previous examples, is critical in order to determine good lower and upper bounds for the MFPT from $n_{\textrm{J}}=\text {S}_{\text {tot}}$ to $n_{\textrm{W}}=\text {S}_{\text {tot}}$.

Let us now determine the lower and upper bounds for the MFPT from $n_{\textrm{J}}=\text {S}_{\text {tot}}$ to $n_{\textrm{W}}=\text {S}_{\text {tot}}$. In particular, here we have

5.14 $$\begin{aligned} G_{+}=\{1,2,3,4\}\;\;\;\textrm{and}\;\;\;G_{-}=\emptyset , \end{aligned}$$

and the rate of increase $\lambda _z({\check{x}})$ and the rate of decrease $\gamma _z({\check{x}})$ can be written as

5.15 $$\begin{aligned} \lambda _z({\check{x}})=f_1({\check{x}})+f_2({\check{x}})+f_3({\check{x}})+f_4({\check{x}})\;\;\;\textrm{and}\;\;\;\gamma _z({\check{x}})=0, \end{aligned}$$

with $f_1({\check{x}})$, $f_2({\check{x}})$, $f_3({\check{x}})$, $f_4({\check{x}})$ defined in (5.12).

The two continuous time Markov chains $$\breve{X}$$ and $$ \overset{\frown}{\mathit{X}}$$, are defined on the same state space as $${\check{X}}$$ and have infinitesimal generators $${\breve{Q}}$$ and $$ \overset{\frown}{\mathit{Q}}$$, respectively, such that, for $$z\in \{\ell , \ell +1 ,\dots , u-1,u\}$$ and $${\check{x}}\in \mathcal {L}_z$$, $${\breve{Q}}_{{\check{x}},{\check{x}}+\check{v}_k} = \frac{\lambda ^M_z}{|G_{+} ({\check{x}})|}$$ for $$k \in G_{+} ({\check{x}})$$ and $$ \overset{\frown}{\mathit{Q}}_{{\check{x}},{\check{x}}+\check{v}_k} = \frac{\lambda ^m_z}{|G_{+} ({\check{x}})|}$$ for $$k \in G_{+} ({\check{x}})$$, with $$\lambda ^{M}_z=\max _{{\check{x}}\in \mathcal {L}_z}\lambda _z({\check{x}})$$ and $$\lambda ^{m}_z=$$$$\min _{{\check{x}}\in \mathcal {L}_z}\lambda _z({\check{x}})$$, as defined in (4.4), and non-empty $$G_{+} ({\check{x}})$$ defined as in (4.5) where $$G_{+}$$ is given in (5.14).

Then, as described in Sect. 4.3, we can compare the Markov chain ${\check{X}}$ with $\breve{X}$ and $\overset{\frown}{\mathit{X}}$, separately, to obtain analytical expressions for lower and upper bounds for the MFPT from $n_{\textrm{J}}=\text {S}_{\text {tot}}$ to $n_{\textrm{W}}=\text {S}_{\text {tot}}$, which can be written asrespectively. Since

$$\lambda ^{M}_z=\max _{{\check{x}}\in \mathcal {L}_z}\lambda _z({\check{x}}) = \max _{{\check{x}}\in \mathcal {L}_z} \left( f_1({\check{x}})+f_2({\check{x}})+f_3({\check{x}})+f_4({\check{x}})\right), $$

and

$$\lambda ^{m}_z=\min _{{\check{x}}\in \mathcal {L}_z}\lambda _z({\check{x}})=\min _{{\check{x}}\in \mathcal {L}_z}\left( f_1({\check{x}})+f_2({\check{x}})+f_3({\check{x}})+f_4({\check{x}})\right) $$

we observe that lower and upper bounds $\breve{h}_{\ell ,u}$ and $\overset{\frown}{\mathit{h}}_{\ell ,u}$ for the MFPT under consideration decrease as any of the rate constants $k_1,k_2,k_3,k_4$ increases. This result suggests that, if any reaction associated with either pathway in the bi-parallel network becomes faster, then the upper and lower bounds for the mean time needed to transform all J into all W (i.e., the MFPT from $n_{\textrm{J}}=\mathrm {S_{tot}}$ to $n_{\textrm{W}}=\mathrm {S_{tot}}$) decrease.

We note that, by following the same approach used above, these results can be generalized to any network having two species (J and W) connected by n-parallel pathways (beyond just two).

## Conclusion

In this paper, we started by providing a description of Stochastic Chemical Reaction Networks (SCRNs), which are a class of continuous time Markov chain models commonly used to describe the stochastic behavior of chemical reaction systems (Sect. 2). We then introduced the notion of *coclique level structure* (Sect. 3) and developed theoretical tools for identifying such coclique level structures for continuous time Markov chains associated with SCRNs, where each reaction involves the consumption of one molecule of a given species and the production of one molecule of another species, and an associated graph $\mathcal {G}$ is weakly connected (Sect. 4.1). Additionally, we provided conditions for identifying if a SCRN does or does not admit a coclique level structure (Sect. 4.1). Finally, we derived analytical expressions for upper and lower bounds for MFPTs of SCRNs having a coclique level structure (Sect. 4.3).

Following this, we provided illustrative examples to demonstrate the utility of our theoretical tools in studying the stochastic behavior of SCRNs (Sect. 5). More precisely, we focused on models describing the main interactions among histone modifications alone, and in combination with DNA methylation (Bruno et al. 2022a), as well as on a bi-parallel network motif, a typical building block found in complex networks (Milo et al. 2002). Through these examples, we demonstrated that our algorithm for identifying coclique level structures is easy to apply, and the analytical expressions for upper and lower bounds of MFPTs obtained with our theoretical tools provide mechanistic insights into how system parameters affect the stochastic behavior of SCRNs. This mechanistic insight is particularly valuable for applications where it is crucial to understand which biological parameters must be tuned to modulate system dynamics in a specific manner.

The mathematical results and theoretical tools developed in this paper can be applied to all stochastic models that meet the considered assumptions. Future work will be focused on generalizing these results by relaxing some of these assumptions, such as allowing the Markov chain to have countably many states and allowing SCRNs to have more than one molecule consumed and produced per reaction. Another valuable direction for future work is the implementation of the algorithm introduced in Sect. 4.2. While in this paper we focused on providing theoretical guidance, a practical implementation of the algorithm would broaden the applicability of our approach and facilitate its integration into simulation pipelines.

## Supplementary Information

Below is the link to the electronic supplementary material.

Supplementary file 1 (pdf 484 KB)

## Data Availability

Data sharing not applicable to this article as no datasets were generated or analysed during the current study.

## Footnotes

1 In this stoichiometric matrix, there are no duplicate reaction vectors. This parallels combining reaction intensity functions associated with the same reaction vector as in (2.2).

2 If one starts with the chemical reactions associated with a SCRN, for example as in (5.1), one can form the associated complex-reaction graph, also known as the Feinberg-Horn-Jackson graph, and compute the deficiency associated with that graph. This graph need not be the same as our graph $\mathcal {G}$, and its deficiency need not necessarily be zero, for example as in (5.1).

3 Note that we allow two vertices connected by edges $e$ and the reverse of $e$ to be a wd-cycle.

4 Here, $S^q$ is a $d_q\times r_q$ matrix, where $r_q$ is the number of edges in $\mathcal {G}^q$. A component $\mathcal {G}^q$ of $\mathcal {G}$ consisting of one vertex has no edges and the associated $S^q$ is degenerate.

5 With this reduction, coordinates corresponding to graph components consisting of a single vertex are removed, although the transition rates of the Markov chain ${\check{X}}$ may depend on the constant amount of any species associated with such an isolated vertex.

## References

[CR1] Anderson DF, Kurtz TG (2015) Stochastic Analysis of Biochemical Systems. Springer, Berlin

[CR2] Backenköhler M, Bortolussi L, Wolf V (2020) *Bounding mean first passage times in population continuous-time Markov chains*. In: QEST 2020: 17th international conference on quantitative evaluation of systems, vol 12289, pp 155–174

[CR3] Brglez F, Bryan D, Koiminski K (1989) Combinational profiles of sequential benchmark circuits. IEEE Int Symp Circuits Syst Public Lib Sci 3:1929–1934

[CR4] Bruno S, Williams RJ, Del Vecchio D (2022) Epigenetic cell memory: the gene’s inner chromatin modification circuit. PLoS Comput Biol Public Lib Sci 18(4):1–27 10.1371/journal.pcbi.1009961 PMC8985953 35385468

[CR5] Bruno S, Williams RJ, Del Vecchio D (2022) Model reduction and stochastic analysis of the histone modification circuit. European Control Conference (ECC) 2022:264–271

[CR6] Bruno S, Williams RJ, Del Vecchio D (2023) Mathematical analysis of the limiting behaviors of a chromatin modification circuit. Math Control Signals Syst 35:399–432

[CR7] Bruno S, Campos FA, Fu Y, Del Vecchio D, Williams RJ (2024) Analysis of singularly perturbed stochastic chemical reaction networks motivated by applications to epigenetic cell memory. SIAM J Appl Dyn Syst 23(4):2695–2731

[CR8] Campos FA, Bruno S, Fu Y, Del Vecchio D, Williams RJ (2023) Comparison theorems for stochastic chemical reaction networks. Bull Math Biol 85:39 37000280 10.1007/s11538-023-01136-5 PMC10066174

[CR9] Dodd IB, Micheelsen MA, Sneppen K, Thon G (2007) Theoretical analysis of epigenetic cell memory by nucleosome modification. Cell 129(4):813–822 17512413 10.1016/j.cell.2007.02.053

[CR10] Feinberg M (1986) Chemical reaction network structure and the stability of complex isothermal reactors—I. The Deficiency Zero and deficiency one theorems. Chem Eng Sci 42(10):2229–2268

[CR11] Gaver DP, Jacobs PA, Latouche G (1984) Finite birth-and death models in randomly changing environments. Adv Appl Probab 16:715–731

[CR12] Haseltine EL, Rawlings JB (2002) Approximate simulation of coupled fast and slow reactions for stochastic chemical kinetics. J Chem Phys 117:6959–6969

[CR13] Kang HW, KhudaBukhsh WR, Koeppl H, Rempala GA (2019) Quasi-steady-state approximations derived from the stochastic model of enzyme kinetics. Bull Math Biol 81:1303–1336 30756234 10.1007/s11538-019-00574-4

[CR14] Kelsey V, Roney-Dougal CM (2022) Maximal cocliques in the generating graphs of the alternating and symmetric groups. Combin Theory. 10.48550/arXiv.2007.12021

[CR15] Kleinberg J, Tardos E (2005) Algorithm design, 1st ed. Pearson Education, Inc

[CR16] Lopatatzidis S, De Bock J, de Cooman G (2017) Computing lower and upper expected first-passage and return times in imprecise birth–death chains. Int J Approx Reason 80(2):137–173

[CR17] Milo R, Shen-Orr S, Itzkovitz S, Kashtan N, Chklovskii D, Alon U (2002) Network motifs: simple building blocks of complex networks. Science 298(5594):824–7 12399590 10.1126/science.298.5594.824

[CR18] Norris JR (1997) Markov chains. Cambridge University Press, Cambridge

[CR19] Srivastava R, You L, Summers J, Yin J (2002) Stochastic versus deterministic modeling of intracellular viral kinetics. J Theor Biol 218(3):309–321 12381432 10.1006/jtbi.2002.3078

[CR20] van Mieghem P (2010) Graph spectra for complex networks. Cambridge University Press, Cambridge

[CR21] Wilson RJ (1996). *Introduction to Graph Theory.* 4th ed., Addison Wesley Longman

