# Supplementary material for: Coclique level structure for stochastic chemical reaction networks
Source: J Math Biol. 2025 Nov 10;91(6):78. doi: 10.1007/s00285-025-02261-6 (PMC12602679; doi:10.1007/s00285-025-02261-6)
Supplement: Supplementary file 1 — (pdf 484 KB) [file 285_2025_2261_MOESM1_ESM.pdf]

Coclique Level Structure for Stochastic Chemical Reaction Networks

Simone Bruno[1,2,*], Yi Fu[3,*], Felipe A. Campos[4], Domitilla Del Vecchio[2], and Ruth J. Williams[4]

[1]*Department of Data Science, Dana-Farber Cancer Institute, 450 Brookline Avenue, Boston, MA 02115. Email:* `sbruno@ds.dfci.harvard.edu`

[2]*Department of Mechanical Engineering, Massachusetts Institute of Technology, 77 Massachusetts Avenue, Cambridge, MA 02139. Email:* `ddv@mit.edu`

[3]*Bioinformatics and Systems Biology Program, University of California, San Diego, 9500 Gilman Drive, La Jolla CA 92093-0112. Email:* `yif064@ucsd.edu`

[4]*Department of Mathematics, University of California, San Diego, 9500 Gilman Drive, La Jolla CA 92093-0112. Email:* `(fcamposv,rjwilliams)@ucsd.edu`

[*]*These authors contributed equally: S. Bruno and Y. Fu*

Supplementary Information (SI)

S.1 Proof of Lemma 3.1

Before introducing the proof of Lemma 3.1, we provide some definitions used in the proof. The **degree** of a vertex is the number of edges that are incident to the vertex, where we count both incoming and outgoing edges. A **weakly directed tree** is a directed graph whose underlying undirected graph is a tree. Given a directed graph with d vertices, a **weakly directed spanning tree** is a subgraph of the graph with all d vertices and such that it is a weakly directed tree, and it will have $d-1$ edges. We abbreviate weakly directed spanning tree as wd-spanning tree. Please note that a weakly connected graph $\mathcal{G}$ has a wd-spanning tree.

Proof. Consider a wd-spanning tree of the graph $\mathcal{G}$. Let $\widehat{\mathcal{V}}_{st} = \{\widehat{v}_1, \widehat{v}_2, \ldots, \widehat{v}_{d-1}\}$ denote the set of $d-1$ reaction vectors associated with the wd-spanning tree edges. We first show that the stoichiometric matrix associated with $\widehat{\mathcal{V}}_{st}$, $S_{d,d-1} = [\widehat{v}_1, \widehat{v}_2, \ldots, \widehat{v}_{d-1}] \in \mathbb{Z}^{d\times(d-1)}$ has rank $d-1$. We prove this by induction. First consider $d=2$. In this case the only possible wd-spanning tree is given by the two vertices and one edge connecting them. This means that $\text{rank}(S_{2,1}) = 1$. Then, let us assume that the result is true for any wd-spanning tree with $d-1$ vertices (i.e., $\text{rank}(S_{d-1,d-2}) = d-2$) for some $d \geq 3$, and consider a wd-spanning tree with d vertices. This wd-spanning tree always has a degree-one vertex, that we define as *vertex* 1, and then we can rearrange the rows and columns of $S_{d,d-1}$ so that

$$S_{d,d-1} = \begin{bmatrix} \pm 1 & 0 \\ * & S_{d-1,d-2} \end{bmatrix}.$$

Then, $S_{d-1,d-2}$ is the stoichiometric matrix associated with the wd-spanning tree with the *vertex* 1 and associated edge removed. By the induction hypothesis, we can conclude that $S_{d-1,d-2}$ has rank $d-2$, and then $\text{rank}(S_{d,d-1}) = d-1$. Since $S_{d,d-1}$ is obtained by removing columns associated with the reaction vectors $v_k \in \mathcal{V} \setminus \widehat{\mathcal{V}}_{st}$ from S, this result implies that $\text{rank}(S) \geq d-1$.

Under Assumption 3.1, $\mathbb{1}^T v_k = 0$ for $k = 1, \ldots, n$, and thus $\mathbb{1}^T S = 0$. This means that $\mathbb{1} \in \ker(S^T)$ and then $\dim\ker(S^T) \geq 1$. By the rank-nullity theorem (see for example [3]), we have that $\mathrm{rank}(S) = \mathrm{rank}(S^T) = d - \dim\ker(S^T) \leq d - 1$. Putting together the results obtained, we can conclude that $\mathrm{rank}(S) = d - 1$.

Furthermore, given that $\mathbb{1}^T S = 0$ and $\dim\ker(S^T) = 1$, then $\mathbb{1}$ is the only (up to scalar multiplication) conservation vector such that $S^T \mathbb{1} = 0$. □

S.2 Proof of Theorem 4.4

Let $\breve{S}^q \in \mathbb{Z}^{(d_q-1)\times n}$ be the first $(d_q - 1)$ rows of the stoichiometric matrix S^q. Then, similar to (3.7) and since the stoichiometric matrix for the SCRN has the form (4.17), the system (4.21) can be re-written in matrix-vector form as

$$\begin{bmatrix} \left(\breve{S}^1\right)^T & 0 & 0 \\ 0 & \ddots & 0 \\ 0 & 0 & \left(\breve{S}^p\right)^T \end{bmatrix} b = w, \tag{S.1}$$

which is equivalent to

$$\left(\breve{S}^q\right)^T b^q = w^q \qquad \text{for every } q = 1, \ldots, p \text{ such that } |\mathcal{G}^q| > 1,$$

where for $q = 1, \ldots, p$, the vectors $b^q, w^q \in \mathbb{Z}^{d_q-1}$ are the qth entries of $b = (b^1, \ldots, b^p)^T$ and $w = (w^1, \ldots, w^p)^T$, respectively. Note that for $q = 1, \ldots, p$, if $|\mathcal{G}^q| = 1$, then $\breve{S}^q \in \mathbb{Z}^{(d_q-1)\times n}$ is a $0 \times n$ matrix, which does not appear in (S.1), and $\breve{x}^q$ is a zero-dimensional vector, and so we do not need to consider that component in any coclique level function, as in (4.22). Consider $q = 1, \ldots, p$ such that $|\mathcal{G}^q| > 1$. As noted before Theorem 4.4, there is a SCRN associated with each $\mathcal{G}^q$. The stoichiometric matrix for this SCRN is S^q. By Theorem 4.1, $\left(\breve{S}^q\right)^T b^q = w^q$ has a solution $b^q \in \mathbb{Z}^{d_q-1}$ if and only if $L^q : \mathbb{Z}^{d_q-1} \to \mathbb{Z}$ given by $L^q(\breve{x}^q) = (b^q)^T \breve{x}^q$ is a coclique level function for the SCRN associated with $\mathcal{G}^q$. Thus, L is a coclique level function for the whole SCRN if and only if

$$L(\breve{x}) = b^T \breve{x} = (b^1, \ldots, b^p)^T (\breve{x}^1, \ldots, \breve{x}^p) = \sum_{\substack{q=1,\ldots,p: \\ |\mathcal{G}^q|>1}} (b^q)^T \breve{x}^q = \sum_{\substack{q=1,\ldots,p: \\ |\mathcal{G}^q|>1}} L^q(\breve{x}^q),$$

where L^q is a coclique level function for the SCRN associated with $\mathcal{G}^q$ where $|\mathcal{G}^q| > 1$.

For each weakly connected component $\mathcal{G}^q$ such that $|\mathcal{G}^q| > 1$, by Theorem 4.3, there exists a coclique level structure for the SCRN associated with $\mathcal{G}^q$ if and only if $\mathcal{G}^q$ is bipartite. Since $\mathcal{G}$ is bipartite if and only if each $\mathcal{G}^q$ with $|\mathcal{G}^q| > 1$ is bipartite, we conclude that a coclique level function for $\breve{X}$ exists if and only if $\mathcal{G}$ is bipartite. □

S.3 One-dimensional birth-death process: Mean first passage time.

Let us consider a one-dimensional irreducible finite state continuous time Markov chain in which the state space $\mathcal{X} = \{0, 1, \ldots, K\}$ and the off-diagonal entries of the infinitesimal generator Q are all zero except for the following positive rates:

$$\begin{aligned} Q_{x,x+1} &= \lambda_x \quad \text{if } x \in \{0, \ldots, K-1\}, \\ Q_{x,x-1} &= \gamma_x \quad \text{if } x \in \{1, \ldots, K\}. \end{aligned} \tag{S.2}$$

In other words, Q is the infinitesimal generator for a finite state birth-death process.

We will determine an analytical expression for the MFPT from $x = K$ to $x = 0$ and from $x = 0$ to $x = K$ for this chain. To this end, it is important to note that X can be equivalently characterized using holding times with exponential parameters $\{q_x\}_{x\in\mathcal{X}}$ and a transition probability matrix P for the *embedded discrete time Markov chain*. More precisely, for each $x \in \mathcal{X}$, $q_x = -Q_{x,x} \neq 0$, since X is irreducible, and for all $x, y \in \mathcal{X}$, $P_{x,x} = 0$, $P_{x,y} = \frac{Q_{x,y}}{q_x}$, for $y \neq x$ in $\mathcal{X}$. Note that $Q = \text{diag}(q)(P - I)$. Defining $\mathcal{B}$ as a nonempty subset of $\check{\mathcal{X}}$ such that $\mathcal{B} \neq \check{\mathcal{X}}$ and using first step analysis (see (3.1) in [18]), we obtain that the MFPT from x to $\mathcal{B}$ can be written as

$$h_{x,\mathcal{B}} = \begin{cases} 0 & \text{if } x \in \mathcal{B} \\ \frac{1}{q_x} + \sum_{y\in\mathcal{X}} P_{x,y} h_{y,\mathcal{B}} & \text{if } x \in \mathcal{B}^c. \end{cases} \tag{S.3}$$

Now, let us first focus on the MFPT from $x = K$ to $x = 0$. In this case $\mathcal{B} = \{0\}$ and then (S.3) can be rewritten as

$$\begin{cases} h_{0,0} = 0, \\ h_{x,0} = \frac{1}{\lambda_x+\gamma_x} + \frac{\lambda_x}{\lambda_x+\gamma_x} h_{x+1,0} + \frac{\gamma_x}{\lambda_x+\gamma_x} h_{x-1,0} & \text{if } x \in \{1, \ldots, K-1\}, \\ h_{K,0} = \frac{1}{\gamma_K} + h_{K-1,0}, \end{cases} \tag{S.4}$$

where for $x, y \in \mathcal{X}$, $h_{x,y} = \mathbb{E}_x[\tau_y]$, $\tau_y = \inf\{t \geq 0 : X(t) = y\}$, X is the continuous time Markov chain with infinitesimal generator given by (S.2). Now, defining $\Delta h_{x,x-1} = h_{x,0} - h_{x-1,0}$ for $x \in \{1, \ldots, K\}$, we can rewrite (S.4) in the following way:

$$\begin{cases} h_{0,0} = 0, \\ \Delta h_{x,x-1} = \frac{1}{\gamma_x} + \frac{\lambda_x}{\gamma_x} \Delta h_{x+1,x} & \text{if } x \in \{1, \ldots, K-1\}, \\ \Delta h_{K,K-1} = \frac{1}{\gamma_K}. \end{cases} \tag{S.5}$$

From (S.5), we have an explicit formula for $\Delta h_{K,K-1}$ and any $\Delta h_{x,x-1}$ can be expressed as a function of $\Delta h_{x+1,x}$. Furthermore, if we sum the $\Delta h_{x,x-1}$ for $x =$

$1, \dots, K$, we obtain

$$h_{K,0} = h_{K,0} - h_{0,0} = \sum_{x=1}^{K} (\Delta h_{x,x-1}) = \Delta h_{1,0} + \Delta h_{2,1} + \cdots + \Delta h_{K-1,K-2} + \Delta h_{K,K-1}. \tag{S.6}$$

Thus, to evaluate the MFPT from $x = K$ to $x = 0$, we can calculate $\Delta h_{x,x-1}$ for $x = K, K-1, \dots, 1$ and then sum all of the terms. We then obtain

$$\begin{aligned} h_{K,0} &= \frac{1}{\gamma_K}\left(1 + \frac{\lambda_{K-1}}{\gamma_{K-1}} + \frac{\lambda_{K-1}\lambda_{K-2}}{\gamma_{K-1}\gamma_{K-2}} + \cdots + \frac{\lambda_{K-1}\dots\lambda_1}{\gamma_{K-1}\cdots\gamma_1}\right) \\ &\quad + \frac{1}{\gamma_{K-1}}\left(1 + \frac{\lambda_{K-2}}{\gamma_{K-2}} + \frac{\lambda_{K-2}\lambda_{K-3}}{\gamma_{K-2}\gamma_{K-3}} + \cdots + \frac{\lambda_{K-2}\dots\lambda_1}{\gamma_{K-2}\cdots\gamma_1}\right) + \cdots + \frac{1}{\gamma_1} \\ &= \frac{1}{\gamma_1} + \sum_{i=2}^{K}\frac{1}{\gamma_i}\left(1 + \sum_{j=1}^{i-1}\frac{\lambda_j\dots\lambda_{i-1}}{\gamma_j\cdots\gamma_{i-1}}\right). \end{aligned} \tag{S.7}$$

With a similar procedure, we can obtain the MFPT from $x = 0$ to $x = K$. More precisely, we have

$$\begin{aligned} h_{0,K} &= \frac{1}{\lambda_0}\left(1 + \frac{\gamma_1}{\lambda_1} + \frac{\gamma_1\gamma_2}{\lambda_1\lambda_2} + \cdots + \frac{\gamma_1\cdots\gamma_{K-1}}{\lambda_1\dots\lambda_{K-1}}\right) \\ &\quad + \frac{1}{\lambda_1}\left(1 + \frac{\gamma_2}{\lambda_2} + \frac{\gamma_2\gamma_3}{\lambda_2\lambda_3} + \cdots + \frac{\gamma_2\cdots\gamma_{K-1}}{\lambda_2\dots\lambda_{K-1}}\right) + \cdots + \frac{1}{\lambda_{K-1}} \\ &= \frac{1}{\lambda_{K-1}} + \sum_{i=0}^{K-2}\frac{1}{\lambda_i}\left(1 + \sum_{j=i+1}^{K-1}\frac{\gamma_{i+1}\cdots\gamma_j}{\lambda_{i+1}\dots\lambda_j}\right). \end{aligned} \tag{S.8}$$

A more detailed derivation of the $h_{0,K}$ and $h_{K,0}$ is given in [1].

S.4 Theorems 3.3 and 3.4 from [2]

Let A be an $m \times d$ matrix, with no rows identically zero, and $K_A = \{y \in \mathbb{R}^d : Ay \geq 0\}$. For $x, y \in \mathbb{R}^d$, we say that $x \preccurlyeq_A y$ whenever $A(y - x) \geq 0$. For a non-empty set $\Gamma \subseteq \mathcal{X} \subseteq \mathbb{Z}_+^d$, we say that a set Γ is increasing in $\mathcal{X}$ with respect to $\preccurlyeq_A$ if for every $x \in \Gamma$ and $y \in \mathcal{X}$, $x \preccurlyeq_A y$ implies that $y \in \Gamma$. Moreover, we say that a set $\Gamma \subseteq \mathcal{X}$ is decreasing in $\mathcal{X}$ with respect to $\preccurlyeq_A$ if for every $x \in \Gamma$ and $y \in \mathcal{X}$, $y \preccurlyeq_A x$ implies that $y \in \Gamma$. Furthermore, for $x \in \mathbb{R}^d$, let $K_A + x = \{y \in \mathbb{R}^d : x \preccurlyeq_A y\}$ and $\partial_i(K_A + x) := \{y \in K_A + x : \langle A_{i\bullet}, y\rangle = \langle A_{i\bullet}, x\rangle\}$[6] for each $1 \leq i \leq m$. We can then

[6] Here, for convenience of notation, let $A_{i\bullet}$ denote the row vector corresponding to the i-th row of A, for $1 \leq i \leq m$. In this article we will adopt the convention of considering the inner product $\langle\cdot,\cdot\rangle$ as a function of a row vector in its first entry and as a function of a column vector in the second entry. In particular, $\langle A_{i\bullet}, x\rangle = \sum_{k=1}^{d} A_{ik}x_k$.

characterize the boundary of $K_A + x$ as follows:

$$\partial(K_A + x) = \bigcup_{i=1}^{m} \partial_i(K_A + x). \tag{S.9}$$

Finally, we introduce the concept of usual stochastic order $\preccurlyeq_{st}$ for two random variables Y, Z: we say that Y is smaller than Z in the usual stochastic order, that is, $Y \preccurlyeq_{st} Z$, if $F_Y(t) \geq F_Z(t)$ for every $t \in \mathbb{R}$, where F_Y and F_Z are the cumulative distribution functions for Y and Z, respectively.

In the following theorem, we consider the set of distinct vectors $\{\eta^1, \ldots, \eta^s\}$ formed by Av_j, for $1 \leq j \leq n$, where s denotes the cardinality of this set, and we consider the subsets of indices

$$G^k := \{j : 1 \leq j \leq n \text{ and } Av_j = \eta^k\}, \quad \text{for } 1 \leq k \leq s.$$

The following theorem applies even if $\mathcal{X}$ is countably infinite, although in this paper all of our state spaces are finite.

Theorem S.1 (immediate consequence of Theorem 3.3 in [2][7]). *Consider a non-empty set $\mathcal{X} \subseteq \mathbb{Z}^d_+$, a collection of distinct vectors $v_1, \ldots, v_n$ in $\mathbb{Z}^d \setminus \{0\}$ and two collections of non-negative (intensity) functions on $\mathcal{X}$, $\Upsilon = (\Upsilon_1, \ldots, \Upsilon_n)$ and $\breve{\Upsilon} = (\breve{\Upsilon}_1, \ldots, \breve{\Upsilon}_n)$ such that if $x + v_j \notin \mathcal{X}$, then $\Upsilon_j(x) = \breve{\Upsilon}_j(x) = 0$, and assume the associated continuous time Markov chains (with intensity functions given by Υ and $\breve{\Upsilon}$, respectively, for the transition directions $v_1, \ldots, v_n$) do not explode in finite time. Consider a matrix $A \in \mathbb{Z}^{m \times d}$ with non-zero rows and suppose that both of the following conditions hold:*

(i) For each $1 \leq j \leq n$, the vector Av_j has entries in $\{-1, 0, 1\}$ only.
(ii) For each $x \in \mathcal{X}$, $1 \leq i \leq m$ and $y \in \partial_i(K_A + x) \cap \mathcal{X}$ we have that

$$\sum_{j \in G^k} \breve{\Upsilon}_j(y) \leq \sum_{j \in G^k} \Upsilon_j(x), \quad \textit{for each } k \textit{ such that } \eta^k_i < 0, \tag{S.10}$$

and

$$\sum_{j \in G^k} \breve{\Upsilon}_j(y) \geq \sum_{j \in G^k} \Upsilon_j(x), \quad \textit{for each } k \textit{ such that } \eta^k_i > 0. \tag{S.11}$$

Then, for each pair $x^\circ, \breve{x}^\circ \in \mathcal{X}$ such that $x^\circ \preccurlyeq_A \breve{x}^\circ$, there exists a probability space $(\Omega, \mathcal{F}, \mathbb{P})$ with two continuous time Markov chains $X = \{X(t) : t \geq 0\}$ and $\breve{X} = \{\breve{X}(t) : t \geq 0\}$ defined there, each having state space $\mathcal{X} \subseteq \mathbb{Z}^d_+$, with infinitesimal generators Q and $\breve{Q}$, associated with Υ and $\breve{\Upsilon}$ respectively (as in (2.4)), with initial conditions $X(0) = x^\circ$ and $\breve{X}(0) = \breve{x}^\circ$ and such that:

$$\mathbb{P}\left[X(t) \preccurlyeq_A \breve{X}(t) \textit{ for every } t \geq 0\right] = 1. \tag{S.12}$$

[7] Compared to Theorem 3.3 in [2], Theorem S.1 includes some additional clarifications inserted in parentheses to improve clarity and completeness in the context of this paper.

Theorem S.2 (immediate consequence of Theorem 3.4 in [2][8]). *Consider a non-empty set* $\mathcal{X} \subseteq \mathbb{Z}_+^d$*, a collection of distinct vectors* $v_1, \ldots, v_n$ *in* $\mathbb{Z}^d \setminus \{0\}$ *and two collections of non-negative (intensity) functions on* $\mathcal{X}$*,* $\Upsilon = (\Upsilon_1, \ldots, \Upsilon_n)$ *and* $\breve{\Upsilon} = (\breve{\Upsilon}_1, \ldots, \breve{\Upsilon}_n)$*, such that if* $x + v_j \notin \mathcal{X}$*, then* $\Upsilon_j(x) = \breve{\Upsilon}_j(x) = 0$*, and assume the associated continuous time Markov chains (with intensity functions given by* Υ *and* $\breve{\Upsilon}$*, respectively, for the transition directions* $v_1, \ldots, v_n$*) do not explode in finite time. Consider a matrix* $A \in \mathbb{Z}^{m \times d}$ *with non-zero rows and conditions (i) and (ii) in Theorem S.1 are satisfied.*

Let $x^\circ, \breve{x}^\circ \in \mathcal{X}$ *be such that* $x^\circ \preccurlyeq_A \breve{x}^\circ$ *and let* $X = \{X(t) : t \geq 0\}$ *and* $\breve{X} = \{\breve{X}(t) : t \geq 0\}$ *be two continuous time Markov chains (possibly defined on different probability spaces), each having state space* $\mathcal{X} \subseteq \mathbb{Z}_+^d$*, with infinitesimal generators* Q *and* $\breve{Q}$*, associated with* Υ *and* $\breve{\Upsilon}$ *respectively, and with initial conditions* $X(0) = x^\circ$ *and* $\breve{X}(0) = \breve{x}^\circ$*. For a non-empty set* $\Gamma \subseteq \mathcal{X}$*, consider* $T_\Gamma := \inf\{t \geq 0 : X(t) \in \Gamma\}$ *and* $\breve{T}_\Gamma := \inf\{t \geq 0 : \breve{X}(t) \in \Gamma\}$*. If* Γ *is increasing in* $\mathcal{X}$ *with respect to the relation* $\preccurlyeq_A$*, then*

$$\breve{T}_\Gamma \preccurlyeq_{st} T_\Gamma, \tag{S.13}$$

and the mean first passage time of $\breve{X}$ *from* $\breve{x}^\circ$ *to* Γ *is dominated by the mean first passage time of* X *from* x° *to* Γ*. If* Γ *is decreasing in* $\mathcal{X}$ *with respect to the relation* $\preccurlyeq_A$*, then*

$$T_\Gamma \preccurlyeq_{st} \breve{T}_\Gamma, \tag{S.14}$$

and the mean first passage time of X *from* x° *to* Γ *is dominated by the mean first passage time of* $\breve{X}$ *from* $\breve{x}^\circ$ *to* Γ*.*

The proof of these theorems can be found in Sections 5.3 and 3.3 of [2], respectively.

[8] Compared to Theorem 3.4 in [2], Theorem S.2 includes some additional clarifications inserted in parentheses to improve clarity and completeness in the context of this paper.

References

[1] Bruno, S., Williams, R.J. and Del Vecchio, D. (2022). *Epigenetic cell memory: The gene's inner chromatin modification circuit.* PLOS Computational Biology, Public Library of Science, vol. 18(4): 1 - 27.

[2] Campos, F.A., Bruno, S., Fu, Y., Del Vecchio, D. and Williams, R.J. (2023). Comparison theorems for stochastic chemical reaction networks. *Bull Math Biol*, vol. 85(39).

[3] Friedberg, S. H., Insel, A. J. and Spence, L. E. (2014). *Linear Algebra, 4th edition.* Pearson Education.

[4] Norris, J.R. (1997). *Markov Chains.* Cambridge University Press.
